# Cardiovascular disease incidence rates: a study using routinely collected health data

**DOI:** 10.1186/s40959-023-00189-8

**Published:** 2023-11-15

**Authors:** Johanna Ramroth, Rebecca Shakir, Sarah C. Darby, David J. Cutter, Valerie Kuan

**Affiliations:** 1 Oxford Population Health, University of Oxford, Richard Doll Building, Old Road Campus, Oxford, OX3 7LF UK; 2 Oxford University Hospitals NHS Foundation Trust, Churchill Hospital, Old Road, Oxford, OX3 7LE UK; 3 Institute of Health Informatics, University College London, London, WC1N 1AX UK

**Keywords:** Ischemic heart disease, Heart failure, Valvular heart disease, Stroke, Systemic anti-cancer therapy, Radiotherapy

## Abstract

**Background:**

There is substantial evidence that systemic anticancer therapies and radiotherapy can increase the long-term risk of cardiovascular disease (CVD). Optimal management decisions for cancer patients therefore need to take into account the likely risks from a proposed treatment option, as well as its likely benefits. For CVD, the magnitude of the risk depends on the incidence of the disease in the general population to which the patient belongs, including variation with age and sex, as well as on the treatment option under consideration. The aim of this paper is to provide estimates of CVD incidence rates in the general population of England for use in cardio-oncology and in other relevant clinical, research and health policy contexts.

**Methods:**

We studied a population-based representative cohort, consisting of 2,633,472 individuals, derived by electronic linkage of records from primary care with those of admitted-patient care in England during April 1, 2010, to April 1, 2015. From 38 individual CVDs available via the linked dataset we identified five relevant categories of CVD whose risk may be increased by cancer treatments: four of heart disease and one of stroke.

**Results:**

We calculated incidence rates by age-group and sex for all relevant CVD categories combined, for the four relevant categories of heart disease combined, and for the five relevant CVD categories separately. We present separate incidence rates for all 38 individual CVDs available via the linked dataset. We also illustrate how our data can be used to estimate absolute CVD risks in a range of people with Hodgkin lymphoma treated with chemotherapy and radiotherapy.

**Conclusions:**

Our results provide population-based CVD incidence rates for a variety of uses, including the estimation of absolute risks of CVD from cancer treatments, thus helping patients and clinicians to make appropriate individualized cancer treatment decisions.

**Graphical Abstract:**

Graphical Abstract: Cardiovascular incidence rates for use in cardio-oncology and elsewhere: A presentation of age- and sex-specific cardiovascular disease (CVD) incidence rates for use in calculation of absolute cardiovascular risks of cancer treatments, and in other clinical, research and health policy contexts. Abbreviations – CVD: cardiovascular disease; y: years

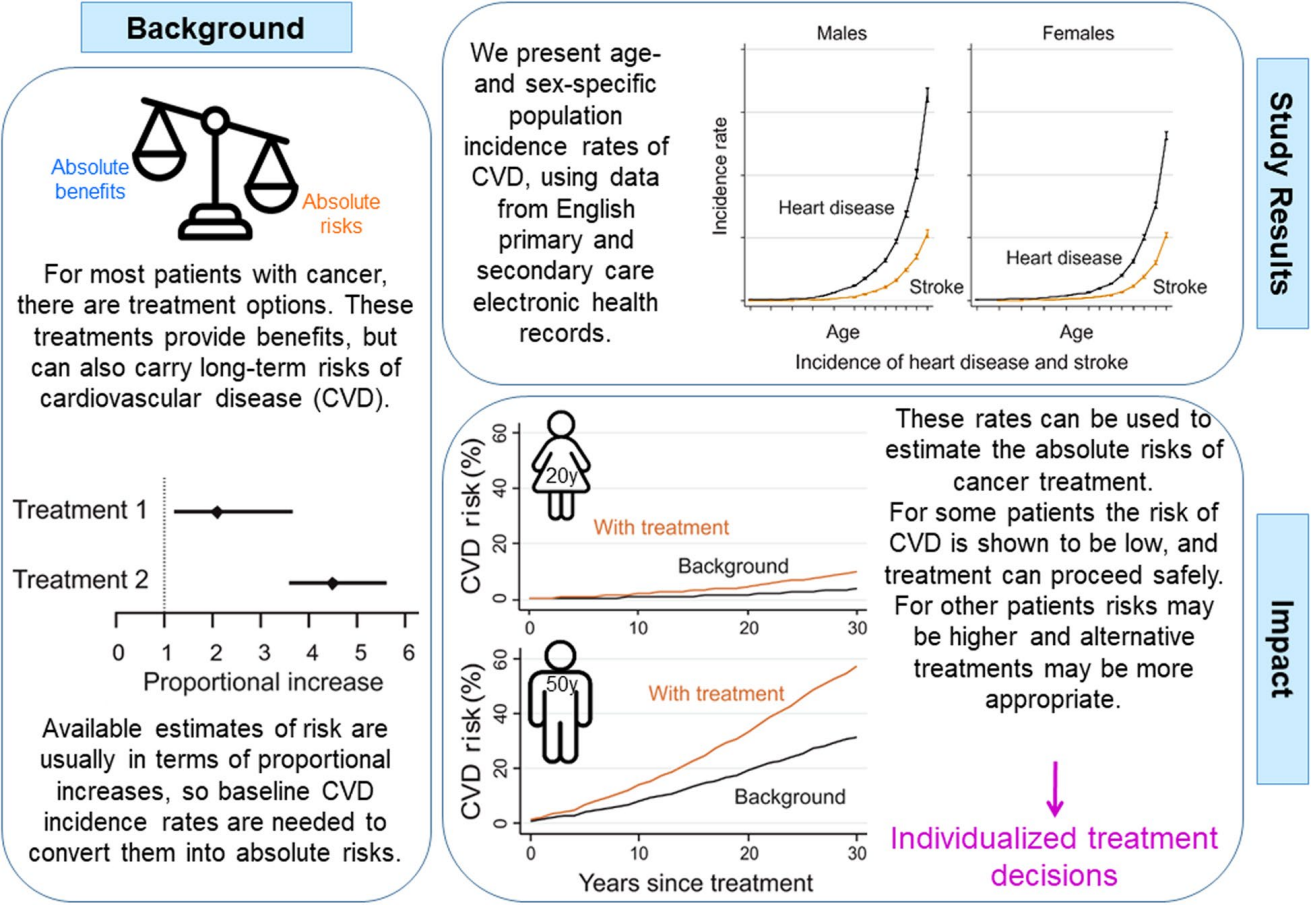

**Supplementary Information:**

The online version contains supplementary material available at 10.1186/s40959-023-00189-8.

## Background

Recent decades have seen substantial improvements in cancer treatments and survival, but there are concerns about the effects of some treatments on the long-term risks of cardiovascular disease (CVD) [1]. Systemic anticancer treatments (SACT), particularly anthracyclines and trastuzumab, are known to induce cardiomyopathy which can lead to heart failure [2]. Radiotherapy to the thorax can increase the risks of atherosclerotic coronary artery disease, valvular heart disease and pericardial disease [3] while radiotherapy to the neck, brain [4] and possibly also to the mediastinum, can increase the risk of ischemic stroke [5]. Screening of patients who have had radiotherapy to the mediastinum has shown a significant proportion have conduction defects [6], although the magnitude of the risk following contemporary doses is currently uncertain.

Individual treatment decisions can only be made on an informed basis if patients and clinicians have information predicting the likely risks as well as the likely benefits of all available treatment options. The majority of information on the long-term risks of CVD resulting from cancer treatment has been provided by epidemiological studies in which large groups of cancer patients, with documented levels of exposure to SACT and/or radiotherapy, have been followed over many years. These studies provide information on the proportional increases in the rates of particular diseases arising after treatment with a certain dose of SACT or radiation [7–10]. In order to be helpful in determining the optimal treatment strategy for individual cancer patients, these proportional estimates need to be combined with background incidence rates and converted into absolute risks. For example, a proportional estimate may suggest that a patient’s incidence rate of ischemic heart disease (IHD) would increase by 30% if they underwent a certain treatment, but if their background IHD rate is already high, that will translate into a much bigger absolute increase in risk than for an individual whose background rate is low. Estimates of the incidence rates of CVDs in general populations are, therefore, instrumental to predicting the likely absolute risks of CVD from cancer treatments. CVD incidence rates vary substantially by age and sex, so, in order to be useful, incidence rates must also be age- and sex-specific.

Here, we report CVD incidence rates in a large, representative, population sample derived from primary care records linked to hospital records in England. We focus on the most clinically relevant CVD categories, that is, those for which it is widely accepted that the risk can be increased by cancer treatments, and we exclude people with a pre-existing diagnosis of a relevant CVD. We provide information on all relevant CVDs combined, all relevant heart diseases combined and five relevant CVD categories separately. We also provide separate incidence rates for all 38 individual CVDs available via the linked dataset we have used.

## Methods

### Study population

We studied pseudonymized population-based electronic health records (EHRs) of primary-care patient-level data from the Clinical Practice Research Datalink (CPRD) linked via NHS numbers, sex and post-code of home address to Hospital Episode Statistics (HES) for admitted-patient care [11, 12]. Individuals were eligible for the study if they met the following criteria on April 1, 2010: aged at least one year; registered with a general practice in England that had consented to linkage and that had contributed up-to-standard data to CPRD for at least one year; and the individual’s record met research standards set by the CPRD [11]. The study was conducted in accordance with the Declaration of Helsinki and approved by the Independent Scientific Advisory Committee for the Medicines and Healthcare products Regulatory Agency (protocol 16_022).

### Identification of relevant CVDs

**Fig. 1:**
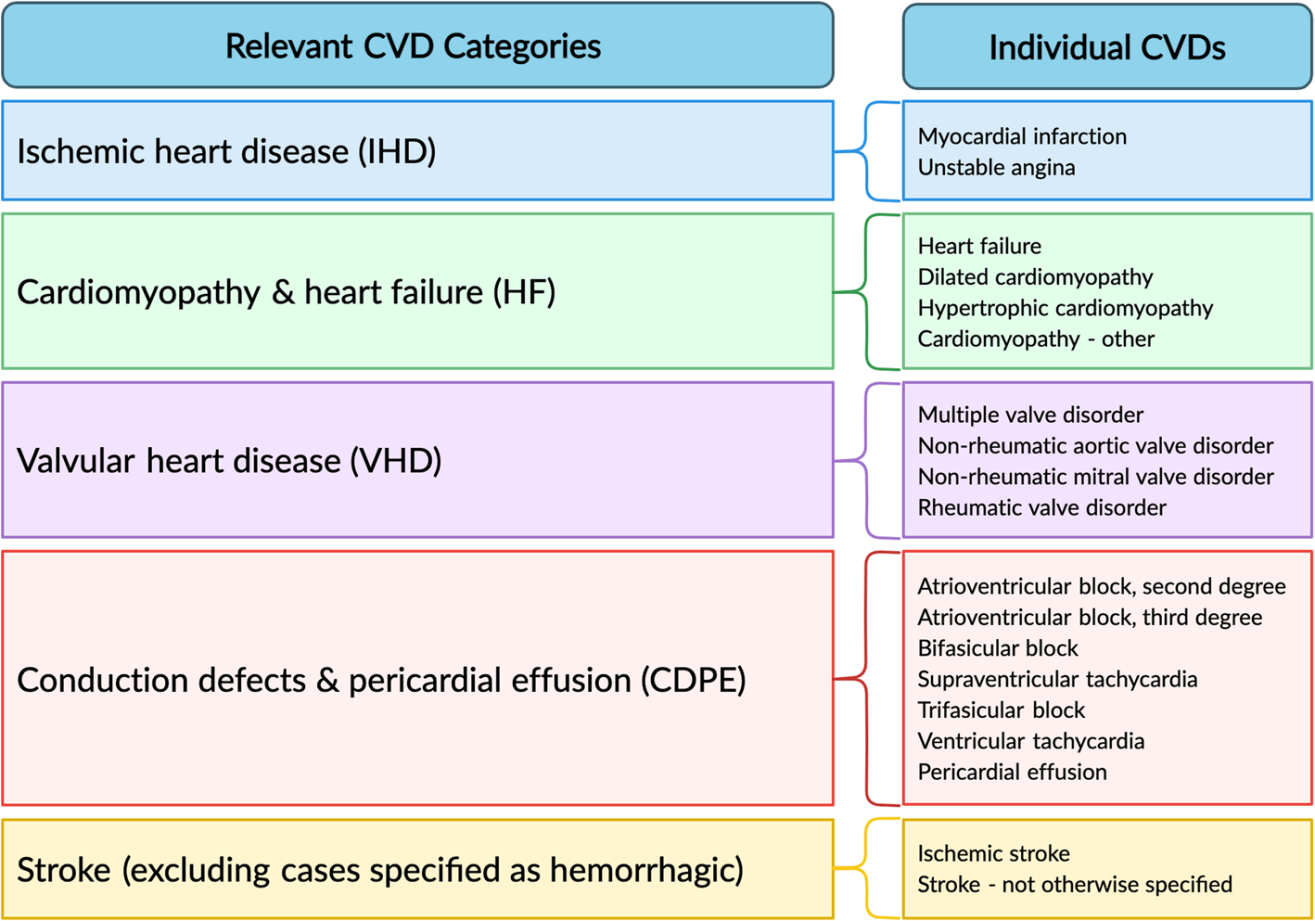
Categorized and individual cardiovascular diseases (CVDs). The five relevant cardiovascular disease categories whose risk can be increased by cancer treatments, and their composition in terms of individual CVDs defined using algorithms from the CALIBER platform. See Additional Table e1 for further details

Case definitions were based on phenotyping algorithms for 38 CVDs obtained from the CALIBER platform [11, 13]. These were derived from diagnostic and procedural codes in the Read Codes, the International Classification of Diseases tenth revision, and the Office of Population Censuses and Surveys Classification of Interventions and Procedures version 4, as recorded in CPRD and HES and described previously [12]. Details are available on the CALIBER platform [11]. Of these 38 CVDs, we identified 19 for which it is widely accepted that cancer treatments may increase the risk. These 19 relevant diseases were the main focus of this study. We grouped them into five categories: ischemic heart disease (IHD), cardiomyopathy and heart failure (HF), valvular heart disease (VHD), conduction defects & pericardial effusion (CDPE), and stroke (excluding cases specified as hemorrhagic) (Fig. 1). The remaining CVD diagnoses were excluded from our main analyses, either because they are not known to be increased following any cancer treatment (e.g. abdominal aortic aneurysm) or because they are likely to be transient and without lasting direct consequence (e.g. transient ischemic attack) (see Additional Table e1 for details).

**Table 1:** Dose-response relationships and organ-specific radiotherapy doses used in Hodgkin lymphoma (HL) example

CVD	Organ at risk	Relationship between radiation dose measure and relevant CVD			Organ-specific doses in HL example (Gy)	
		**Radiation dose measure**	**Model for relative increase in CVD rate**	**Reference**	**Average**	**Range**
Ischaemic heart disease	Whole heart	Mean dose to whole heart (MHD)	RR = 1 + ERR*MHD ERR = 0.20 if age_tx < 28 y ERR = 0.088 if 28 ≤ age_tx ≤ 36 y ERR = 0.042 if age_tx > 36 y	[7]	7.8	0.8–24.0
Valvular heart disease	Heart valves	Weighted sum of mean doses to valves (sumVD)	RR = 1 + e^(−5.02)^ x sumVD x e^(0.075*sumVD)^	[8]	12.6	0.8–33.1
Cardiomyopathy and heart failure	Left ventricle	Mean dose to left ventricle (MLVD)	RR = 1 + e^(−4.12)^ x MLVD x e^(0.06*MLVD)^ + e^0.97^xd_anthra_ d_anthra_ = 1 if 150 mg/m^2^ of anthracyclines are given d_anthra_ = 0 if no anthracyclines are given	[9]	3.3	0.3–20.5
Stroke	Common carotid arteries	Mean dose to common carotid arteries (MDCCA)	RR = 1 + ERR x MDCCA ERR = 0.0684 if age_tx ≤ 20 y ERR = .0513 if 20 < age_tx ≤ 30 y ERR = .0244 if 30 < age_tx ≤ 40 y ERR = .0098 if age_tx > 40 y	[14]	28.3	10.5–38.1
Other cardiovascular diseases	Various	Various	Dose-response relationships are not available for these diseases individually. As an approximation, a weighted average of the above models can be used, with the weights depending on the diseases and exposures under consideration. For this example, an average of the models for IHD and CHF was used	[15]	Average of MHD and MLVD	-

*Abbreviations*: *y* Years, *age_tx* Age at treatment, *RR* Relative rate, i.e. factor by which age- and sex-specific population-based CVD incidence rate is multiplied due to the cancer treatment, *ERR* Excess relative rate per gray, *Gy* Gray

### Statistical analysis

Many patients develop several CVDs and the development of one disease often increases the risk of subsequently developing another. If a cancer patient has already developed a CVD whose risk is known to be increased by a cancer treatment, it is less likely that they will be considered appropriate for a treatment regime that further increases CVD risk. Therefore, in our main analyses we excluded individuals who had been diagnosed with a condition in any one of our five CVD categories before April 1, 2010. All remaining individuals entered the study on April 1, 2010, and contributed to the person-years at risk from that date until the earliest of: first diagnosis of a relevant CVD (using one of the definitions below); death; de-registration from the practice; last data collection from the practice; or March 31, 2015.

A few individuals had more than one relevant CVD diagnosed on the same day. In this case, IHD and stroke took precedence over the other categories. If both IHD and stroke were diagnosed on the same day, 0.5 was allocated to each category. If diagnoses in several CVD categories other than IHD or stroke were recorded on the same day, 0.5 was added to each category if two CVD categories were involved, or 0.33 if three were involved.

For each analysis, the numbers of diagnoses and the corresponding numbers of person-years at risk were tabulated according to sex and attained age in the following groups: 1–9, 10–19, 20–29, 30–39, 40–49, 50–54, 55–59, 60–64, 65–69, 70–74, 75–79, 80–84, and ≥ 85 years. The numbers of diagnoses in each cell of the tabulation were assumed to follow a Poisson distribution, and incidence rates were calculated as the ratio of the number of diagnoses to the corresponding number of person-years. Confidence intervals were derived on the log scale to take account of the constraint that a rate must be greater than zero [16]. Age-standardized incidence rates were also calculated, using the European standard population for males and females [17]. To preserve confidentiality, categories with fewer than ten diagnoses were not reported.

### CVD endpoints

**Fig. 2:**
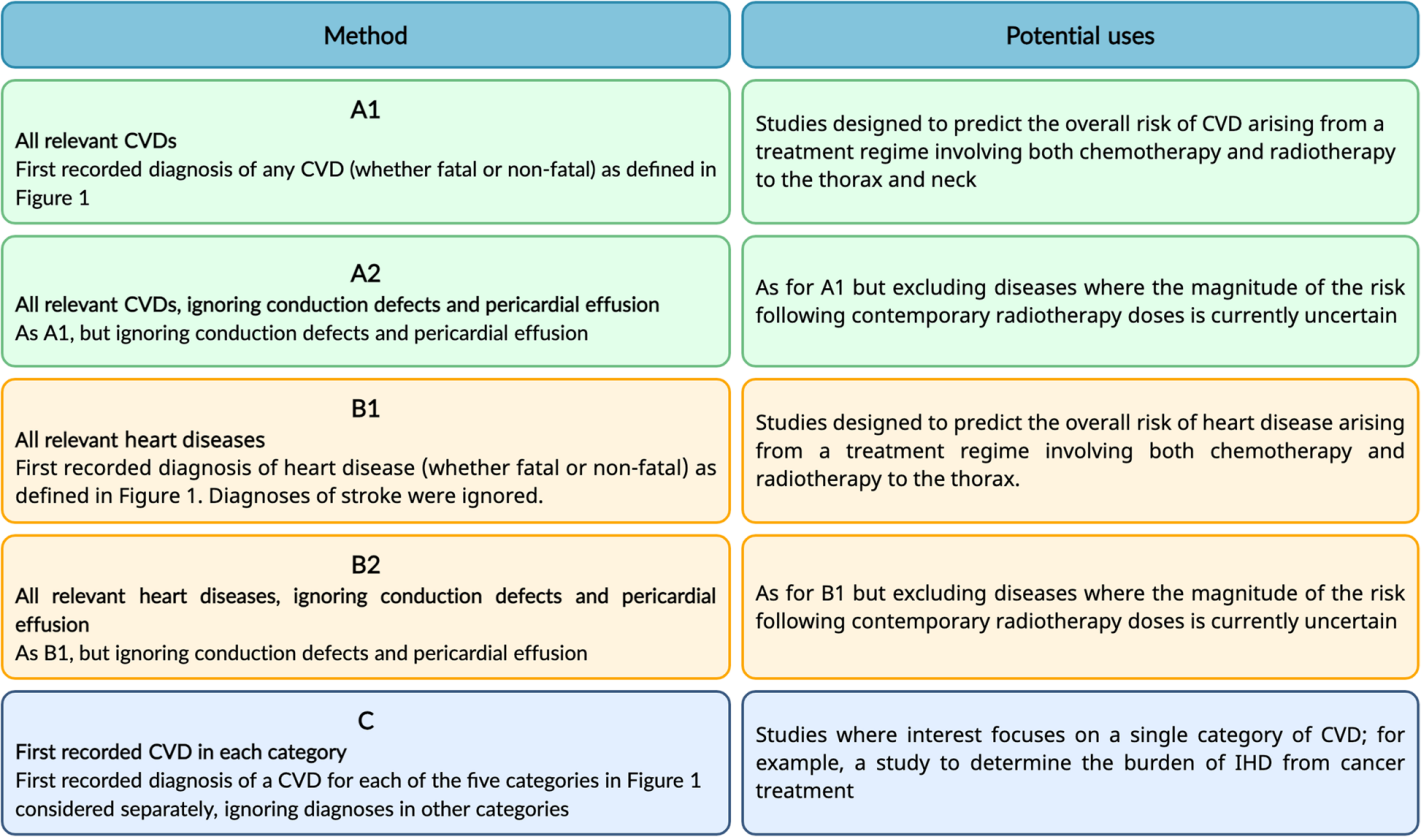
Alternative methods of defining relevant cardiovascular endpoints, and examples of their potential use. CVD: cardiovascular disease; IHD: ischemic heart disease

We calculated incidence rates using a number of different methods to define cardiovascular endpoints of interest. These are described below and summarized in Fig. 2.

First we calculated the incidence rate for all relevant CVD categories combined, considering just the earliest diagnosis of any CVD (Method A1). Some studies investigating radiotherapy regimens that involve treatment of both the heart and neck need to consider both stroke and heart disease, whereas others that consider only thoracic radiotherapy may need to focus just on heart disease. We therefore repeated our calculations considering just diagnoses recorded as heart disease (i.e. IHD, HF, VHD, CDPE) (Method B1); in this calculation, diagnoses recorded as stroke were ignored.

Irrespective of whether a particular study focuses on all CVD or just on heart disease, it may sometimes be appropriate to exclude diseases for which the magnitude of the risk following contemporary doses is currently uncertain, such as CDPE. We therefore repeated our calculations for both all CVD and all heart disease, but ignoring diagnoses recorded as CDPE (Methods A2 and B2).

Sometimes it is of interest to know the contribution that an individual disease category makes to the overall incidence rate. Therefore, for each of the above four methods, we have also provided the contribution of each of the five CVD categories to the overall rate, using just the diagnoses that were attributed to that specific disease category, together with the person-years used in the calculation of the overall rate. At other times, it may be appropriate to consider a single CVD category in isolation (e.g. HF when studying the effect of anthracyclines, or stroke when studying the effect of radiotherapy to the neck). We therefore also calculated incidence rates separately for each of the five categories; for example, for IHD we considered just the first diagnosis in that category and ignored diagnoses in the other four categories. For each category we considered the numbers of person-years appropriate to that specific category (Method C).

In addition to our main analyses, we also calculated incidence rates for all 38 available individual CVDs. The method used was similar to that used for method C of the main analyses except that only individuals who had a diagnosis of the specific disease in question prior to April 1, 2010, were excluded from the rate calculations for that disease.

### Illustrative example for Hodgkin lymphoma treatment

We illustrate how CVD incidence rates can be used to guide cancer treatment decisions by considering the example of early-stage Hodgkin lymphoma (HL). We consider patients who are hypothetical but typical of those randomized in a trial designed to test whether patients with early-stage HL and a complete response on positron-emission tomography (PET) after three standard cycles of chemotherapy with doxorubicin, bleomycin, vinblastine, and dacarbazine (ABVD), require radiotherapy [18]. When the trial was initiated, it had already been established that such patients had excellent 5-year overall survival (> 95%) but it was not known whether the additional benefit of radiotherapy in reducing the risk of a relapse of HL would be outweighed by an increased risk of radiation-related CVD and other possible risks. Patients were therefore randomized to 30 Gy involved field radiotherapy (IFRT) or to no further treatment. Among those randomized to radiotherapy, those with no mediastinal involvement by HL had minimal radiation doses to the whole heart (average mean heart dose 0.3 Gy, range 0.1–1.4 Gy) [15]. In contrast, patients with mediastinal involvement received a mean whole heart dose of 7.8 Gy on average, but varying from 0.8 to 24.0 Gy in individual patients, depending on the size of the radiation fields needed to cover the original extent of the disease and the patient’s individual anatomy (Table 1). Such exposures would result in increased risks of IHD (due to exposure of the whole heart), HF (due to exposure of the left ventricle) and VHD (due to exposure of the cardiac valves). These patients were also at increased risk of ischemic stroke, presumably due to exposure of the common carotid arteries (28.3 Gy on average, varying from 10.5 to 38.1 Gy, depending on the radiation fields used and the patient’s individual anatomy). Estimates of the relationship between the percent increase in the age-specific incidence rate of the relevant CVD per unit radiation dose to the relevant organ for these four diseases have been published and are described in Table 1. In addition, all patients would have been at increased risk of HF due to their chemotherapy, which in this case included 150 mg/m^2^ of anthracycline. An estimate of the relationship between HF and anthracycline dose has also been published (Table 1).

**Fig. 3:**
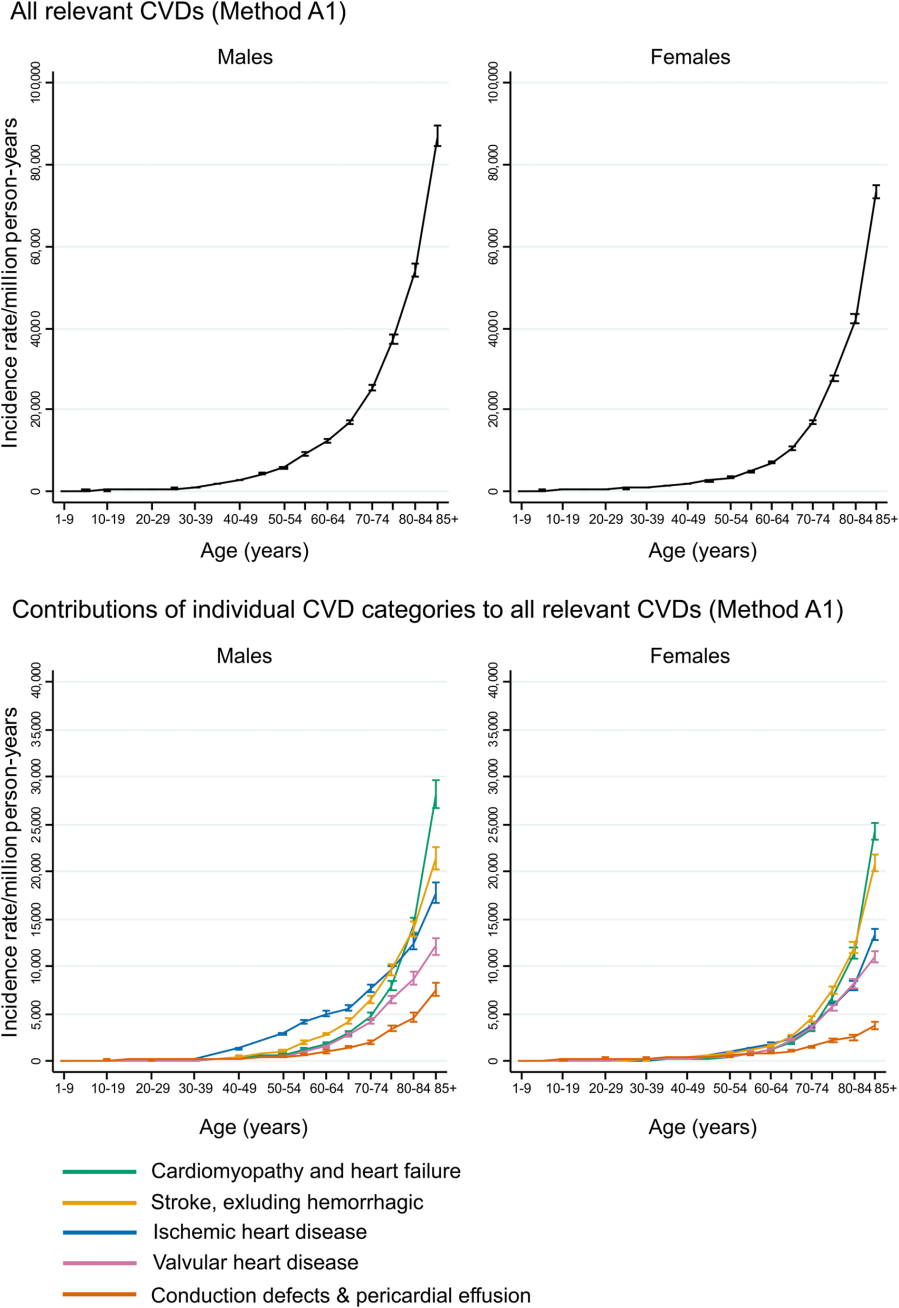
Incidence of all relevant CVDs by age and sex (Method A1). Top row: incidence rate of all relevant CVDs combined, including conduction defects and pericardial effusion, calculated using method A1. Bottom row: contributions of individual CVD categories to incidence rate of all relevant CVDs combined. In all four panels only an individual’s first recorded diagnosis of any relevant CVD is included (see Tables 1 and e2). For each age and sex group, the sum of the rates across the five categories in the bottom row is equal to the corresponding rate for all relevant CVDs in the top row

We considered a series of hypothetical patients, male and female, of different ages, treated for HL. We calculated cumulative risks of any incident CVD over the 30 years following treatment, based on receiving three cycles of ABVD and the average mean organ dose received by patients who had mediastinal radiotherapy in the RAPID trial, taking account of the competing risk of dying from diseases other than CVD. These risks were calculated under three treatment scenarios for each patient: (i) no treatment, (ii) chemotherapy only, and (iii) chemotherapy and radiotherapy. Risks in (i) were calculated using the age- and sex-specific incidence rates for all relevant CVDs, as shown in the top row of Fig. 3 and in Additional Table e2; risks in (ii) were calculated as in (i) but increasing the age-specific rates for HF to allow for the increase in risk from the anthracycline as indicated in Table 2; and in (iii) the risks were calculated as in (ii) but also increasing the age-specific rates for HF, VHD, IHD and other CVDs to take account of the risk from the radiation delivered to the left ventricle, valves, and whole heart respectively. See Additional Text e1 for further details.

**Table 2:** Numbers of individuals in the study and incidence rates for all relevant cardiovascular diseases and all relevant heart diseases, including and excluding events recorded as conduction defects and pericardial effusion, by age and sex

**Age groups (years)**	Numbers of individuals in the study population (%)		**All relevant cardiovascular diseases**				**All relevant heart diseases**			
			**Incidence rate per million person-years (95% confidence interval)**				**Incidence rate per million person-years (95% confidence interval)**			
			**Including conduction defects and pericardial effusion** **(Method A1)**		**Excluding conduction defects and pericardial effusion** **(Method A2)**		**Including conduction defects and pericardial effusion** **(Method B1)**		**Excluding conduction defects and pericardial effusion** **(Method B2)**	
**Females**										
1–9	141 765	(10.8%)	171	*(135-217)*	112	*(84-151)*	151	*(117-194)*	92	*(66-127)*
10–19	154 564	(11.8%)	321	*(279-370)*	141	*(114-175)*	296	*(256-343)*	116	*(92-147)*
20–29	153 845	(11.7%)	621	*(555-695)*	313	*(267-367)*	557	*(494-628)*	249	*(209-298)*
30–39	182 433	(13.9%)	961	*(886-1042)*	617	*(558-683)*	782	*(714-855)*	437	*(387-493)*
40–49	213 879	(16.3%)	1953	*(1859-2051)*	1478	*(1397-1564)*	1542	*(1459-1629)*	1065	*(996-1138)*
50–54	90 825	(6.9%)	3353	*(3175-3541)*	2838	*(2675-3011)*	2549	*(2394-2713)*	2030	*(1893-2178)*
55–59	82 961	(6.3%)	4930	*(4698-5174)*	4179	*(3966-4404)*	3837	*(3633-4052)*	3081	*(2899-3275)*
60–64	85 099	(6.5%)	6999	*(6719-7290)*	6121	*(5860-6393)*	5372	*(5128-5628)*	4496	*(4274-4730)*
65–69	63 521	(4.8%)	10 426	*(10 063-10 801)*	9416	*(9073-9773)*	7944	*(7629-8272)*	6941	*(6647-7247)*
70–74	51 455	(3.9%)	16 975	*(16 438-17 529)*	15 604	*(15 090-16 135)*	12 735	*(12 273-13 214)*	11 319	*(10 885-11 771)*
75–79	40 437	(3.1%)	27 772	*(27 007-28 559)*	25 897	*(25 159-26 657)*	20 631	*(19 976-21 307)*	18 745	*(18 122-19 389)*
80–84	28 671	(2.2%)	42 339	*(41 233-43 475)*	40 039	*(38 966-41 141)*	31 087	*(30 150-32 053)*	28 806	*(27 906-29 735)*
≥ 85	25 893	(2.0%)	73 491	*(71 975-75 039)*	70 258	*(68 780-71 768)*	54 263	*(52 979-55 579)*	51 099	*(49 856-52 373)*
Total individuals	1 315 348	(100.0%)	-	*-*	-	*-*	-	*-*	-	*-*
No. of cases	-		34 037	*-*	30 906	*-*	25 948	*-*	22 762	*-*
Total person-years	-		4 892 483	*-*	4 898 474	*-*	4 905 948	*-*	4 912 064	*-*
Crude rate	-		6957	*(6883-7031)*	6309	*(6239-6380)*	5289	*(5225-5354)*	4634	*(4574-4695)*
Age-standardized rate	-		6935	*(6861-7008)*	6284	*(6214-6354)*	5219	*(5156-5283)*	4568	*(4508-4627)*
**Males**										
1–9	148 578	(11.5%)	219	*(178-269)*	129	*(98-169)*	187	*(150-234)*	97	*(71-133)*
10–19	169 356	(13.1%)	300	*(261-345)*	154	*(127-187)*	279	*(241-322)*	132	*(107-164)*
20–29	161 190	(12.5%)	470	*(416-531)*	285	*(244-333)*	438	*(386-496)*	253	*(214-298)*
30–39	187 354	(14.5%)	920	*(847-1000)*	738	*(673-810)*	786	*(718-859)*	603	*(545-668)*
40–49	221 575	(17.2%)	2881	*(2767-2999)*	2605	*(2497-2718)*	2392	*(2289-2500)*	2116	*(2019-2218)*
50–54	92 305	(7.2%)	5772	*(5537-6017)*	5343	*(5117-5578)*	4724	*(4512-4946)*	4288	*(4087-4500)*
55–59	80 794	(6.3%)	9113	*(8792-9446)*	8475	*(8166-8796)*	7187	*(6903-7483)*	6544	*(6273-6826)*
60–64	78 959	(6.1%)	12 377	*(11 990-12 777)*	11 453	*(11 081-11 837)*	9595	*(9256-9947)*	8670	*(8349-9005)*
65–69	54 989	(4.3%)	17 057	*(16 563-17 567)*	15 747	*(15 272-16 236)*	13 048	*(12 617-13 493)*	11 716	*(11 309-12 138)*
70–74	40 470	(3.1%)	25 296	*(24 568-26 045)*	23 531	*(22 831-24 253)*	19 149	*(18 521-19 799)*	17 339	*(16 742-17 956)*
75–79	27 781	(2.2%)	37 237	*(36 200-38 305)*	34 192	*(33 201-35 213)*	28 069	*(27 177-28 990)*	24 997	*(24 159-25 865)*
80–84	16 496	(1.3%)	54 239	*(52 658-55 867)*	50 360	*(48 843-51 924)*	41 200	*(39 840-42 606)*	37 213	*(35 926-38 545)*
≥ 85	10 492	(0.8%)	87 022	*(84 586-89 530)*	80 493	*(78 162-82 893)*	67 406	*(65 294-69 585)*	60 829	*(58 835-62 890)*
Total individuals	1 290 339	(100.0%)	-	*-*	-	*-*	-	*-*	-	*-*
No. of cases	-		35 404	*-*	32 580	*-*	27 862	*-*	24 946	*-*
Total person-years	-		4 773 726	*-*	4 778 987	*-*	4 787 358	*-*	4 792 811	*-*
Crude rate	-		7416	*(7340-7494)*	6817	*(6744-6892)*	5820	*(5752-5889)*	5205	*(5141-5270)*
Age-standardized rate	-		9603	*(9503-9703)*	8823	*(8727-8918)*	7430	*(7343-7518)*	6641	*(6558-6723)*
**Both sexes combined**										
1–9	290 343	(11.1%)	196	*(167-229)*	121	*(99-147)*	169	*(143-200)*	95	*(76-118)*
10–19	323 920	(12.4%)	310	*(281-343)*	148	*(128-171)*	287	*(259-318)*	125	*(107-146)*
20–29	315 035	(12.1%)	540	*(497-587)*	298	*(267-333)*	493	*(452-538)*	251	*(223-284)*
30–39	369 787	(14.2%)	941	*(887-997)*	678	*(633-726)*	784	*(735-835)*	521	*(481-563)*
40–49	435 454	(16.7%)	2417	*(2343-2493)*	2042	*(1974-2112)*	1967	*(1901-2036)*	1591	*(1531-1653)*
50–54	183 130	(7.0%)	4564	*(4416-4718)*	4092	*(3952-4237)*	3638	*(3506-3775)*	3161	*(3038-3289)*
55–59	163 755	(6.3%)	6999	*(6801-7204)*	6304	*(6115-6498)*	5495	*(5320-5676)*	4795	*(4631-4964)*
60–64	164 058	(6.3%)	9591	*(9354-9835)*	8691	*(8466-8923)*	7410	*(7202-7624)*	6511	*(6316-6711)*
65–69	118 510	(4.5%)	13 532	*(13 299-13 842)*	12 382	*(12 093-12 678)*	10 338	*(10 075-10 609)*	9 182	*(8934-9436)*
70–74	91 925	(3.5%)	20 706	*(20 264-21 159)*	19 160	*(18 735-19 594)*	15 617	*(15 235-16 009)*	14 025	*(13 663-14 395)*
75–79	68 128	(2.6%)	31 766	*(31 141-32 404)*	29 401	*(28 801-30 013)*	23 777	*(23 241-24 326)*	21 392	*(20 885-21 912)*
80–84	45 167	(1.7%)	46 915	*(45 999-47 850)*	44 014	*(43 129-44 917)*	34 987	*(34 205-35 788)*	32 054	*(31 307-32 818)*
≥ 85	36 685	(1.4%)	77 721	*(76 426-79 038)*	73 468	*(72 214-74 745)*	58 381	*(57 275-59 510)*	54 159	*(53 097-55 242)*
Total individuals	2 605 687	(100.0%)	-	*-*	-	*-*	-	*-*	-	*-*
No. of cases	-		69 441	*-*	63 485	*-*	53 810	*-*	47 708	*-*
Total person-years	-		9 666 209	*-*	9 677 461	*-*	9 693 306	*-*	9 704 874	*-*
Crude rate	-		7184	*(7131-7238)*	6560	*(6509-6611)*	5551	*(5505-5598)*	4916	*(4872-4960)*
Age-standardized rate	-		8101	*(8041-8161)*	7412	*(7354-7470)*	6181	*(6129-6233)*	5488	*(5438-5537)*

## Results

### Population characteristics

The study population included 2,633,472 individuals, comprising 5.0% of the total English population. Overall 50.5% were female, with slightly more males in younger age categories, and substantially more females in older ones (Table 2). Thirty-four thousand females were diagnosed with a cardiovascular disease during 4.9 million person-years of observation and 35 thousand males were diagnosed with a cardiovascular disease during 4.8 million years of observation.

### All relevant CVDs (Methods A1 & A2)

The incidence rate for all relevant CVDs rose steeply with age in both males and females (Table 2, Fig. 3 top row). The age-standardized rate was higher in males than in females (males: 9603 per million person-years (mpy), females: 6935 mpy), but the difference between the male and female rates varied by age. At ages up to 40 the rates in the two sexes differed little but, from age 40, the rate was higher in males than in females and the absolute difference between the two increased with increasing age (Additional Figure e1). Rates of each of the component CVDs also increased steeply with age for both sexes. CDPE was the largest individual contributor in the youngest age-groups (Additional Table e2). Then, from age 30 in males and age 50 in females, IHD was the largest contributor (Fig. 3 bottom row). The IHD rate among males increased rapidly at ages 30–55, then more slowly at ages 55–65, before increasing rapidly again among older males. From ages 75 in males and 65 in females IHD was superseded by stroke. From ages 80 in males and 85 in females, HF was the largest contributor.

When events recorded as CDPE were excluded, the age-standardized rates reduced slightly, to 8823 per mpy in males and 6284 per mpy in females (Table 2). The age-specific CVD rates were unchanged at younger ages and reduced slightly at older ages in both males and females (Additional Figures e2, e3 top row, Additional Table e3).

**Table 3:** 30-year cumulative risks (%) of developing incident CVD for example early-stage Hodgkin lymphoma patients, by sex, age at treatment, and type of treatment

Age at treatment (years)	**30-year cumulative risks of incident CVD (%)**					
	MALES			FEMALES		
	Treatment Type			Treatment Type		
	None	CT Only	CT & RT	None	CT Only	CT & RT
20	4.6	7.3	12.4	3.7	6.4	10.0
30	10.9	16.3	23.6	7.1	11.6	16.1
40	20.6	31.3	38.4	13.6	22.1	26.6
50	31.7	49.9	57.6	24.8	41.3	46.8
60	35.9	59.3	67.5	32.9	56.8	64.0

*CT* Chemotherapy (in this case three cycles of ABVD, including 150 mg/m^2^ of anthracyclines), *CT & RT* Chemotherapy and radiotherapy

CVD rates were obtained using Method A1 and are shown in Fig. 3 and Table e2. Competing risks of death from causes other than CVD were assumed to be equal to those in the general population and were taken into account. Further details are given in Additional Text e1

### All relevant heart diseases (Methods B1 & B2)

**Fig. 4:**
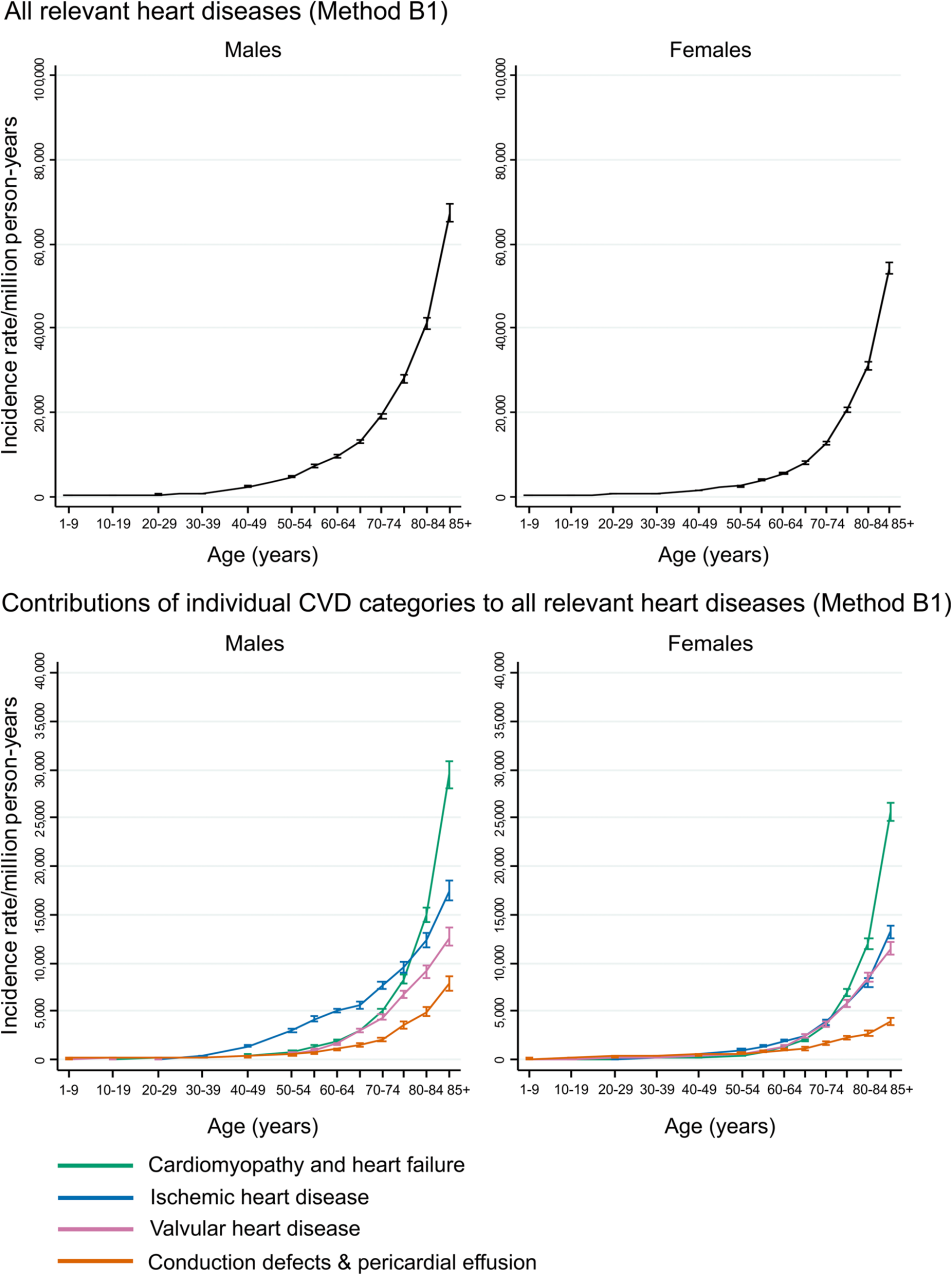
Incidence rate of all relevant heart diseases, by age and sex (Method B1). Top row: incidence rate for all relevant heart diseases combined, including conduction defects and pericardial effusion, calculated using Method B1. Bottom row: the contributions of individual heart disease categories to the incidence rate of all relevant heart diseases combined. In all four panels only an individual’s first recorded diagnosis of any relevant heart disease is included (see Tables 1 and e4). For each age and sex group, the sum of the rates across the four categories in the bottom row is equal to the corresponding rate for all relevant heart diseases in the top row

The incidence rate of all relevant heart diseases rose steeply with age in both males and females (Table 2, Fig. 4 top row) and the age-standardized rate was higher in males (7430 per mpy) than in females (5219 per mpy). At ages under 40, rates were similar in males and females but, above age 40, the rate in males was higher than in females by an increasing absolute amount with increasing age. Among the individual component heart diseases, CDPE made the largest contribution up to age 30 in males and 50 in females (Additional Table e4). Then, IHD made the largest contribution up to age 74 in both males and females, while from age 75, HF made the largest contribution in both sexes (Fig. 4 bottom row).

When events recorded as CDPE were excluded, the age-standardized rates reduced to 6641 per mpy in males and 4568 per mpy in females. Up to age 40 the age-specific rates were similar in the two sexes but, above that, the male rate exceeded the female rate by an increasing amount with increasing age (Table 2, Additional Figure e4 top row). The contributions of the individual component diseases to the overall heart disease rate, after the exclusion of CDPE, were similar to when CDPE was included (Additional Figure e4 bottom row, Additional Table e5).

### Individual categories of CVD (Method C)

**Fig. 5:**
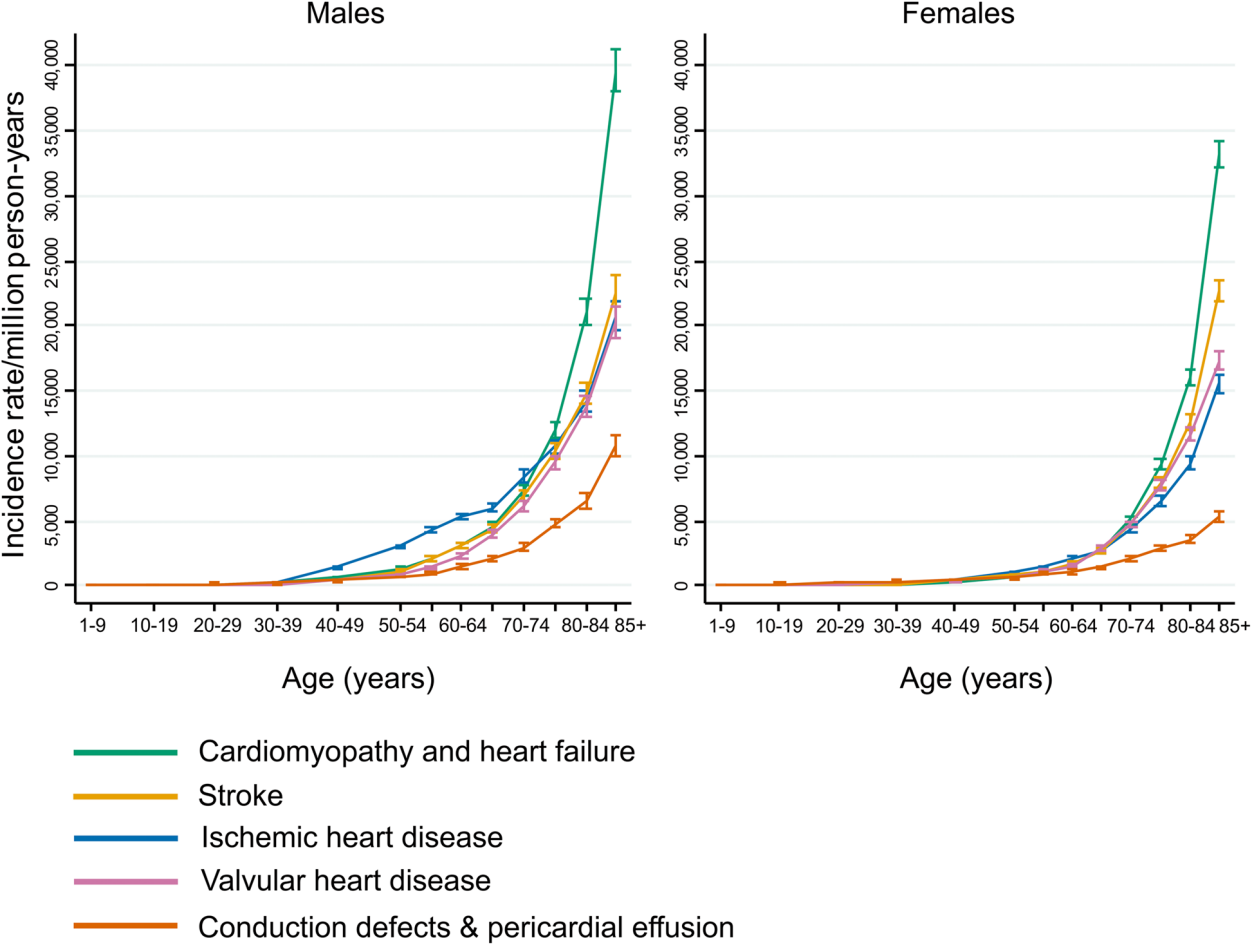
Incidence rate for each of the five relevant CVD categories considered separately, calculated using Method C. The first recorded diagnosis within each CVD category is included, and diagnoses in other CVD categories are ignored (see Table e6). For each age and sex group, the sum of rates across the five categories is usually bigger than the corresponding rate for all relevant CVDs calculated for each using Method A1

Incidence rates considering each of the five relevant CVD categories separately, and ignoring diagnoses in the other four categories, are shown in Fig. 5 and Additional Table e6. The incidence rate for each individual category is always larger than the corresponding contribution to the rate for all relevant CVDs. The CVD categories with the largest proportional increases were VHD, where the age-standardized rate increased by 51% (from 1427 to 2154 per mpy) in males and by 38% (from 1292 to 1777 per mpy) in females and HF, where the age-standardized rate increased by 49% (from 2127 to 3162 per mpy) in males and by 39% (from 1650 to 2297 per mpy) in females. The age-standardized rates for CDPE also increased substantially, by 39% (from 883 to 1228 per mpy) in males and by 25% (from 712 to 888 per mpy) in females. These large increases indicate that in the general population VHD, HF and CDPE often occur secondarily to a previous CVD diagnosis. In contrast, the age-standardized rates for IHD increased by only 9% in males (from 2857 to 3114 per mpy) and by 13% in females (from 1454 to 1637 per mpy), while the age-standardized rates for stroke increased by only 6% in both males and females (males: from 2309 to 2437 per mpy, females: from 1828 to 1937 per mpy) (Additional Tables e2 and e6), indicating that these diseases rarely arise as a consequence of a previous CVD.

Incidence rates for all 38 available individual CVDs available are presented in Additional Table e7.

### Illustrative example for Hodgkin lymphoma treatment

**Fig. 6:**
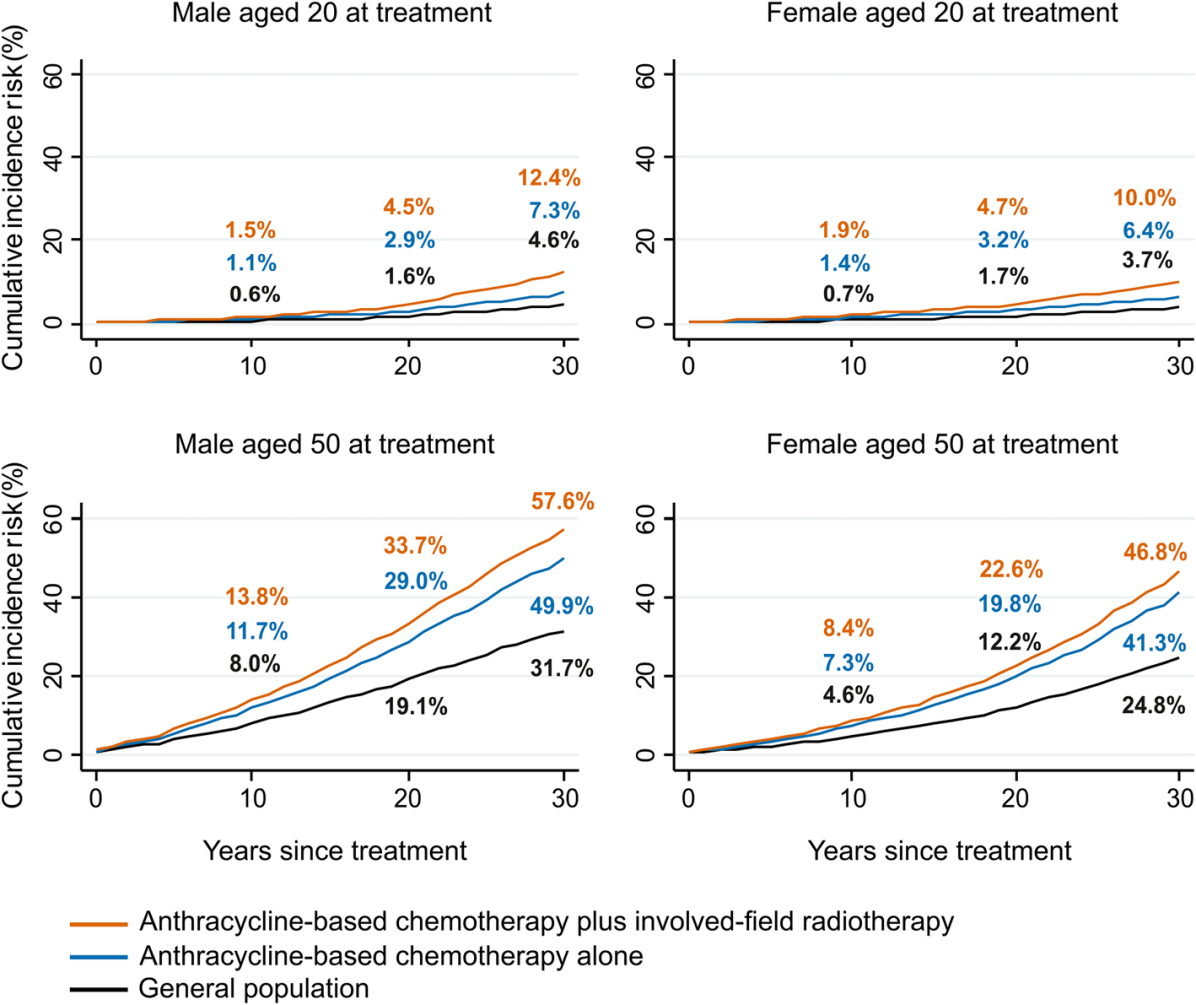
30-year cumulative risks (%) of developing incident CVD for example early-stage Hodgkin lymphoma patients, by sex, age at treatment, and type of treatment. The standard treatment for early-stage Hodgkin lymphoma is anthracycline-based chemotherapy. In addition, the areas of disease may be treated by radiotherapy. The figure shows the cumulative risks of CVD for four example patients receiving identical treatments for Hodgkin lymphoma [19]. The only difference between the patients is their age at treatment and their sex. The decision that the oncologist must make for individual patients is whether the increased risk of CVD is outweighed by the added benefit of the radiotherapy for disease control. CVD rates were obtained using Method A1 and are shown in Fig. 3 and Table e2. Competing risks were taken into account. Further details are given in Additional Text e1

A typical woman in England aged 20 years has a cumulative risk of developing CVD over the next 30 years of 3.7% in the absence of any cancer treatment (Fig. 6). If she receives anthracycline-containing chemotherapy similar to that given to all patients in the RAPID trial this increases by 2.7% to 6.4%. If she also receives radiotherapy with mean doses to her whole heart, left ventricle, heart valves and common carotid arteries, equal to the average doses received by patients randomized to radiotherapy in the RAPID trial, then her risk increases by a further 3.6% to 10.0%. For a man of the same age, the 30-year risks are slightly higher: 4.6% without any cancer treatment, increasing by 2.7% to 7.3% with three cycles of ABVD chemotherapy, and by a further 5.1% to 12.4% with radiotherapy. For older patients the absolute risks are much higher. For a woman aged 50 at diagnosis of HL, her risk of developing a relevant CVD over the next 30 years is 24.8% without any cancer treatment. This increases by 16.5% with anthracycline chemotherapy to 41.3% and by a further 5.5% to 46.8% with radiotherapy. For a man aged 50 at diagnosis, the corresponding 30-year risk with no cancer treatment is 31.7%, increasing by 18.2% with anthracycline to 49.9%, and by a further 7.7% to 57.6% with radiotherapy. Risks for patients irradiated at other ages are given in Table 3. These estimates of CVD risk ignore any mortality risk from HL itself, as opposed to its treatment. For early-stage HL the risk of dying from the disease itself would be small but would need to be taken into account as a competing risk on an individual basis, together with the treatment benefits and their CVD and other risks, e.g. radiation-related cancers, when making individual treatment decisions. The excess risks due to the radiotherapy treatment itself would also vary widely from person to person, given that the dose received to organs at risk ranges widely between individuals.

## Discussion

Recent improvements in cancer treatments have been accompanied by a growing awareness that the benefits they incur may be reduced, and in extreme cases even outweighed, by the risks that certain treatments carry. In particular, there is growing recognition that cancer treatments can substantially increase the risk of CVD, leading to morbidity and, in some cases, early mortality. Epidemiological studies have demonstrated increases in the rates of a range of CVDs in cancer survivors. The results of these studies are usually reported in terms of proportional rather than absolute increases, as proportional changes tend to be more stable than absolute ones across characteristics such as age, sex, geographical region and calendar year. However, proportional increases cannot be used directly to estimate absolute risks of cardiotoxicity from cancer treatments, as the absolute risk depends on both the relevant background CVD incidence rates and the proportional increases that will arise from the cancer treatment. For example, a doubling in the CVD rate would mean that for a young patient whose background risk of developing CVD over the next 30 years is about four percent, the risk would increase to close to eight percent, whereas for an older patient whose background risk of developing CVD in the next 30 years is around 30%, a doubling of the CVD rate would mean a much larger absolute increase in CVD risk to as much as 60%. In order to inform individual treatment decisions for cancer patients, absolute increases in CVD risk must be estimated by combining the age- and sex-specific incidence rates for the relevant CVDs for a population similar to that to which the patient belongs with the proportional increases in those rates obtained from epidemiological studies.

We present background incidence rates from an English population for all categories of CVD whose risk is widely accepted to be increased by current cancer treatments. We have calculated them in a number of different ways, to make them relevant to a wide variety of different situations. We present rates of the relevant CVDs combined, in broad categories, and as individual diseases. Age- and sex-specific rates are reported, along with crude and age-standardized totals.

One example of how our data can be used is in treatment decisions for patients with cancer, where both the individualized benefits and risks of alternative management options must be considered. Randomized trials can be used in conjunction with disease-specific recurrence and mortality rates to estimate the benefits of a particular treatment. Such trials are, however, of limited use for estimating the absolute magnitude of the risks of CVD from treatment. This is partly because reporting of cardiotoxicity in trials may be incomplete, compounded by the lag — often more than a decade — between the completion of cancer treatment and the onset of cardiotoxicity [3], such that conventional trial follow-up may be insufficient to monitor for incident CVDs. Also, there is substantial variability in CVD rates between different populations (e.g. by age, sex and geography) and different calendar periods. In addition, patients enrolled in randomized trials may be healthier on average than patients excluded from the trial and patients treated in previous decades may have a higher background risk of CVD than those treated more recently.

Our data can be used to predict absolute CVD risk, and therefore address the limitations of randomized trials. In the example we compute the thirty-year risks of any CVD based on the age- and sex-specific rates obtained in this study, using Method A1 (Fig. 6, Table 2) for a series of male and female patients of different ages. We then compare each risk with the thirty-year risk for a patient of the same age and sex who has received treatment for HL involving 150 mg/m^2^ of anthracycline-containing chemotherapy and also with the thirty-year risk for a patient who has received radiotherapy (average dose for those with mediastinal involvement) as well as chemotherapy. The treatment-related increases are proportional to the background CVD rate. Therefore, thirty years after receiving treatment, the absolute risks of treatment are higher for males than for females and much higher the older the patient was at the time of treatment. Absolute risks calculated in this way can be compared with the likely absolute benefit from a cancer treatment. The absolute benefit can be calculated by considering the difference in the risk of cancer recurrence or mortality with and without the treatment under consideration, usually using data from randomized trials. Such calculations can also be repeated for multiple cancer treatment options, allowing clinicians and patients to assess the risks of each and compare them with the benefits, together with a patient’s individual characteristics. Situations in which it is critical to be able to weigh up the benefits and risks in this way include the addition of radiotherapy to chemotherapy in the treatment of high-grade non-Hodgkin lymphoma, the use of adjuvant chemotherapy following breast cancer surgery, and the use of varying chemotherapy regimens or the decision between proton-beam therapy and conventional photon radiotherapy to treat various tumor types [15, 19–21].

We have illustrated how our data can be used to predict absolute risks of cardiotoxicity from cancer treatment, when combined with published results on cardiotoxicity from epidemiological studies. However, the data we present are not limited to use in cardio-oncology, and there are many other circumstances in which they may be useful, including health policy, health economics, epidemiology, and informing clinical trial power calculations.

A strength of our study is that we present incidence rates taken from a large, population-based sample. Mortality rates for CVDs by age and sex are available for many countries [22] but reliable nationwide CVD incidence rates are available for only a few. In the UK, incidence rates that combine information from primary and secondary care have recently been published for heart failure [23], but publications for other CVDs or CVD as a whole either include only individuals aged over 30 years, or focus on hospital admissions, prevalence or disability adjusted life years, or do not provide detailed age-specific rates [24–27]. In the UK, more than 98% of the population is registered to receive primary care from a National Health Service (NHS) general practice [11]. Our study sample is derived from these registrations and includes diagnoses in both primary care and in hospital, thus forming one of the largest EHR databases in the world. It has been shown to be representative of the English population by age, sex, and ethnicity [11, 28] and has previously been validated for epidemiological research [11]. CVDs were not included if they were likely to be asymptomatic, or to have only a limited impact on the patient’s quality of life, equivalent to grade 2 or less on the Common Terminology for Adverse Events system [29].

Despite the strengths of our study, it also has limitations. The most obvious of these is that the CVD incidence rates we present do not take into account any cardiovascular risk factors that the patient may have, such as smoking, hypertension or pre-existing cardiovascular disease. Where the effect of such a risk factor — including pre-existing cardiovascular disease — can be estimated, it can be taken into account, at least approximately, by multiplying the relevant incidence rate by the effect of the risk factor and so deriving background CVD rates that are specific to the patient in question. Previous studies have shown that the proportional increase in CVD rate incurred by the cancer treatment is likely to be similar [30]. Where the effect of the risk factor is uncertain, then calculations could be made to demonstrate the highest and lowest likely effects. Our study is also limited by the fact that our incidence rates are specific to England. For a few other countries, such as the United States, Sweden, and the Netherlands, it may be possible to obtain adequate CVD incidence rates from registries or a combination of registry and survey data [31–34]. But for most countries such CVD incidence data are not readily available. Until such time as incidence data do become available for these countries, an approximate solution may be to derive the ratio of the relevant cardiovascular mortality rates to those of England and then to multiply the incidence rates presented here by that factor. Additional limitations specific to the CALIBER platform are discussed elsewhere [11, 12].

## Conclusions

This paper provides clinicians, researchers and policy-makers with representative rates of a wide range of important CVDs from a large English population, using several different calculation methods to suit a variety of analytic needs. We have demonstrated how our data can be used to estimate the absolute risks of cancer treatments, which can facilitate progress in personalized medicine by integrating individualized risk estimation into treatment decisions.

### Supplementary Information

**Additional file 1:** **Additional Table e1.** Classification of the 38 cardiovascular diagnoses available on the CALIBER platform for use in cardio-oncology.** Additional Text e1.** Methods used to predict CVD risks in four example Hodgkin lymphoma patients. **Additional Figure e1.** Incidence rates for all relevant CVDs, by age and sex, and including conduction defects and pericardial effusion (Method A1). **Additional Figure e2.** Incidence rates for all relevant cardiovascular diseases, including and excluding events recorded as conduction defects and pericardial effusion, by age. **Additional Figure e3.** Incidence rate of all relevant CVDs, ignoring conduction defects and pericardial effusion, by age and sex (Method A2). **Additional Figure e4.** Incidence rate of all relevant heart disease, ignoring conduction defects and pericardial effusion, by age and sex (Method B2). **Additional Table e2.** Contributions of each of the five categories to incidence rates of all relevant CVDs (Method A1). **Additional Table e3.** Contributions of each of the four categories to incidence rates of all relevant CVDs, ignoring conduction defects and pericardial effusion (Method A2). **Additional Table e4.** Contributions of each of the four categories to incidence rates of all relevant heart diseases (Method B1). **Additional Table e5.** Contributions of each of the three categories to incidence rates of all relevant heart diseases, ignoring CDPE (Method B2). **Additional Table e6.** Incidence rates for each of the five cardiovascular disease categories, calculated separately (Method C). **Additional Table e7.** Incidence rates of 38 individual cardiovascular diseases, by age and sex.

## Data Availability

Phenotyping algorithms and codelists for all 38 conditions included in our study are publicly available on the CALIBER Portal. They can be downloaded in a machine-readable CSV format from a github data repository for readers to adopt and adapt for their own research. Access to CPRD data, including UK Primary Care Data, and linked data such as Hospital Episode Statistics, is subject to protocol approval via CPRD’s Research Data Governance Process [35].

## Abbreviations

CDPE: Conduction defects & pericardial effusion
CPRD: Clinical Practice Research Datalink
CVD: Cardiovascular disease
EHRs: Electronic health records
HES: Hospital Episode Statistics
HF: Cardiomyopathy and heart failure
IHD: Ischemic heart disease
NHS: National Health Service
SACT: Systemic anticancer treatments
VHD: Valvular heart disease

## References

[1] Herrmann J, Lenihan D, Armenian S, Barac A, Blaes A, Cardinale D (2022). Defining cardiovascular toxicities of cancer therapies: an International Cardio-Oncology Society (IC-OS) consensus statement. Eur Heart J.

[2] Dent SF, Botros J, Rushton M, Aseyev O, Levine MN, Parulekar WR (2020). Anthracycline-induced cardiotoxicity in patients with early-stage breast cancer: the Canadian Cancer Trials Group (CCTG) MA.21 experience. Breast Cancer Res Treat.

[3] Aleman BMP, Van Den Belt-Dusebout AW, De Bruin ML, Van’t Veer MB, Baaijens MHA, De Boer JP (2007). Late cardiotoxicity after treatment for Hodgkin lymphoma. Blood.

[4] Reulen RC, Guha J, Bright CJ, Henson KE, Feltbower RG, Hall M (2021). Risk of cerebrovascular disease among 13 457 five-year survivors of childhood cancer: a population-based cohort study. Int J Cancer.

[5] Gujral DM, Chahal N, Senior R, Harrington KJ, Nutting CM (2014). Radiation-induced carotid artery atherosclerosis. Radiother Oncol.

[6] Adams MJ, Lipsitz SR, Colan SD, Tarbell NJ, Treves ST, Diller L (2004). Cardiovascular status in long-term survivors of Hodgkin’s disease treated with chest radiotherapy. J Clin Oncol.

[7] van Nimwegen FA, Schaapveld M, Cutter DJ, Janus CPM, Krol ADG, Hauptmann M (2015). Radiation dose-response relationship for risk of coronary heart disease in survivors of Hodgkin lymphoma. J Clin Oncol.

[8] Cutter DJ, Schaapveld M, Darby SC, Hauptmann M, van Nimwegen FA, Krol ADG, et al. Risk for valvular heart disease after treatment for Hodgkin lymphoma. JNCI J Natl Cancer Inst. 2015;107. 10.1093/jnci/djv008. 10.1093/jnci/djv008 PMC4394894 25713164

[9] van Nimwegen FA, Ntentas G, Darby SC, Schaapveld M, Hauptmann M, Lugtenburg PJ (2017). Risk of heart failure in survivors of Hodgkin lymphoma: effects of cardiac exposure to radiation and anthracyclines. Blood.

[10] Maraldo MV, Giusti F, Vogelius IR, Lundemann M, Van der Kaaij MAE, Ramadan S (2015). Cardiovascular disease after treatment for Hodgkin’s lymphoma: an analysis of nine collaborative EORTC-LYSA trials. Lancet Haematol.

[11] Denaxas SC, George J, Herrett E, Shah AD, Kalra D, Hingorani AD (2012). Data Resource Profile: Cardiovascular disease research using linked bespoke studies and electronic health records (CALIBER). Int J Epidemiol.

[12] Kuan V, Denaxas S, Gonzalez-Izquierdo A, Direk K, Bhatti O, Husain S (2019). A chronological map of 308 physical and mental health conditions from 4 million individuals in the English National Health Service. Lancet Digit Heal.

[13] CALIBER n.d. https://www.caliberresearch.org/portal. Accessed 11 March 2022.

[14] De Bruin ML, Dorresteijn LDA, Van’t Veer MB, Krol ADG, Van Der Pal HJ, Kappelle AC (2009). Increased risk of stroke and transient ischemic attack in 5-year survivors of hodgkin lymphoma. J Natl Cancer Inst.

[15] Cutter DJ, Ramroth J, Diez P, Buckle A, Ntentas G, Popova B (2021). Predicted risks of cardiovascular disease following chemotherapy and radiotherapy in the UK NCRI RAPID trial of positron emission tomography-directed therapy for early-stage Hodgkin lymphoma. J Clin Oncol.

[16] Kirkwood BR, Sterne JAC (2003). Essential Medical Statistics.

[17] Revision of the European Standard Population: Report of Eurostat’s task force. 2013. 10.2785/11470.

[18] Radford J, Illidge T, Counsell N, Hancock B, Pettengell R, Johnson P (2015). Results of a trial of PET-directed therapy for early-stage Hodgkin’s lymphoma. N Engl J Med.

[19] Taylor C, Duane FK, Dodwell D, Gray R, Wang Z, Wang Y (2017). Estimating the risks of breast cancer radiotherapy: Evidence from modern radiation doses to the lungs and heart and from previous randomized trials. J Clin Oncol.

[20] Ntentas G, Dedeckova K, Andrlik M, Aznar MC, Shakir R, Ramroth J (2021). Proton therapy in Supradiaphragmatic lymphoma: predicting treatment-related mortality to help optimize patient selection. Int J Radiat Oncol.

[21] Jones DA, Candio P, Shakir R, Ntentas G, Ramroth J, Gray AM (2022). Informing radiotherapy decisions in stage I/IIa Hodgkin lymphoma: modeling life expectancy using radiation dosimetry. Blood Adv.

[22] WHO Mortality Database - WHO n.d. https://www.who.int/data/data-collection-tools/who-mortality-database. Accessed 11 March 2022.

[23] Conrad N, Judge A, Tran J, Mohseni H, Hedgecott D, Crespillo AP (2018). Temporal trends and patterns in heart failure incidence: a population-based study of 4 million individuals. Lancet.

[24] George J, Rapsomaniki E, Pujades-Rodriguez M, Shah AD, Denaxas S, Herrett E (2015). How Does Cardiovascular Disease First Present in Women and Men? Incidence of 12 Cardiovascular Diseases in a Contemporary Cohort of 1,937,360 People. Circulation.

[25] Vardas P, Townsend N, Torbica A, Katus H, De Smedt D, Timmis (Chair Writing Group) A (2022). European Society of Cardiology: cardiovascular disease statistics 2021. Eur Heart J.

[26] Roth GA, Mensah GA, Johnson CO, Addolorato G, Ammirati E, Baddour LM (2020). Global Burden of Cardiovascular Diseases and Risk Factors, 1990–2019: Update From the GBD 2019 Study. J Am Coll Cardiol.

[27] Heart & Circulatory Disease Statistics 2022 | BHF n.d. https://www.bhf.org.uk/what-we-do/our-research/heart-statistics/heart-statistics-publications/cardiovascular-disease-statistics-2022. Accessed 11 March 2022.

[28] Mathur R, Bhaskaran K, Chaturvedi N, Leon DA, Van Staa T, Grundy E (2014). Completeness and usability of ethnicity data in UK-based primary care and hospital databases. J Public Health (Bangkok).

[29] Cancer Institute N (2017). Common Terminology Criteria for Adverse Events (CTCAE) v5.0.

[30] Darby SC, Ewertz M, McGale P, Bennet AM, Blom-Goldman U, Brønnum D (2013). Risk of ischemic heart disease in women after radiotherapy for breast cancer. N Engl J Med.

[31] Berry D (2009). SWEDEHEART: Sweden’s new online cardiac registry, the first of its kind. Eur Heart J.

[32] Savarese G, Vasko P, Jonsson Å, Edner M, Dahlström U, Lund LH (2019). The Swedish Heart Failure Registry: a living, ongoing quality assurance and research in heart failure. Ups J Med Sci.

[33] Amsterdam UMC Locatie AMC - NHR – National Heart Registry n.d. https://www.amc.nl/web/research-75/departments/medical-informatics-1/medical-informatics-1/nhr-national-heart-registry.htm. Accessed 10 March 2022.

[34] Berry JD, Dyer A, Cai X, Garside DB, Ning H, Thomas A (2012). Lifetime Risks of Cardiovascular Disease. N Engl J Med.

[35] Data access | CPRD n.d. https://cprd.com/data-access. Accessed 11 July 2023.

