# Supplementary material for: Cardiovascular disease incidence rates: a study using routinely collected health data
Source: Cardiooncology. 2023 Nov 15;9:41. doi: 10.1186/s40959-023-00189-8 (PMC10647140; doi:10.1186/s40959-023-00189-8)
Supplement: Supplementary file 1 — Additional file 1: Additional Table e1. Classification of the 38 cardiovascular diagnoses available on the CALIBER platform for use in cardio-oncology. Additional Text e1. Methods used to predict CVD risks in four example Hodgkin lymphoma patients. Additional Figure e1. Incidence rates for all relevant CVDs, by age and sex, and including conduction defects and pericardial effusion (Method A1). Additional Figure e2. Incidence rates for all relevant cardiovascular diseases, including and excluding events recorded as conduction defects and pericardial effusion, by age. Additional Figure e3. Incidence rate of all relevant CVDs, ignoring conduction defects and pericardial effusion, by age and sex (Method A2). Additional Figure e4. Incidence rate of all relevant heart disease, ignoring conduction defects and pericardial effusion, by age and sex (Method B2). Additional Table e2. Contributions of each of the five categories to incidence rates of all relevant CVDs (Method A1). Additional Table e3. Contributions of each of the four categories to incidence rates of all relevant CVDs, ignoring conduction defects and pericardial effusion (Method A2). Additional Table e4. Contributions of each of the four categories to incidence rates of all relevant heart diseases (Method B1). Additional Table e5. Contributions of each of the three categories to incidence rates of all relevant heart diseases, ignoring CDPE (Method B2). Additional Table e6. Incidence rates for each of the five cardiovascular disease categories, calculated separately (Method C). Additional Table e7. Incidence rates of 38 individual cardiovascular diseases, by age and sex. [file 40959_2023_189_MOESM1_ESM.pdf]

Additional Material

Contents

Additional Methods 2

Additional Table e1: Classification of the 38 cardiovascular diagnoses available on the CALIBER platform for use in cardio-oncology. 2

Additional Text e1: Methods used to predict CVD risks in four example Hodgkin lymphoma patients 3

Additional Results: Figures 5

Additional Figure e1: Incidence rates for all relevant CVDs, by age and sex, and including conduction defects and pericardial effusion (Method A1) 5

Additional Figure e2: Incidence rates for all relevant cardiovascular diseases, including and excluding events recorded as conduction defects and pericardial effusion, by age 6

Additional Figure e3: Incidence rate of all relevant CVDs, ignoring conduction defects and pericardial effusion, by age and sex (Method A2) 7

Additional Figure e4: Incidence rate of all relevant heart disease, ignoring conduction defects and pericardial effusion, by age and sex (Method B2) 8

Additional Results: Tables 9

Additional Table e2: Contributions of each of the five categories to incidence rates of all relevant CVDs (Method A1) 9

Additional Table e3: Contributions of each of the four categories to incidence rates of all relevant CVDs, ignoring conduction defects and pericardial effusion (Method A2) 11

Additional Table e4: Contributions of each of the four categories to incidence rates of all relevant heart diseases (Method B1) 13

Additional Table e5: Contributions of each of the three categories to incidence rates of all relevant heart diseases, ignoring CDPE (Method B2) 15

Additional Table e6: Incidence rates for each of the five cardiovascular disease categories, calculated separately (Method C) 17

Additional Table e7: Incidence rates of 38 individual cardiovascular diseases, by age and sex 19

References 37

Additional Methods

Additional Table e1: Classification of the 38 cardiovascular diagnoses available on the CALIBER platform for use in cardio-oncology.

Diagnoses were excluded from our main analyses if they were not known to be increased following any cancer treatment (e.g. abdominal aortic aneurysm) or because they are likely to be transient and without lasting consequences (e.g. transient ischemic attack).

**Ischemic heart disease**	
Inclusions	*Exclusions*
Myocardial infarction	Stable angina
Unstable angina	Coronary heart disease, NOS
**Cardiomyopathy & heart failure**	
Inclusions	
Dilated cardiomyopathy	
Heart failure	
Hypertrophic cardiomyopathy	
Cardiomyopathy, other	
**Valvular heart disease**	
Inclusions	
Multiple valve disorder	
Non-rheumatic aortic valve disorder	
Non-rheumatic mitral valve disorder	
Rheumatic valve disorder	
**Conduction defects & pericardial effusion**	
Inclusions	*Exclusions*
Atrioventricular block, second degree	Atrial fibrillation
Atrioventricular block, third degree	Atrioventricular block, first degree
Bifasicular block	Left bundle branch block
Pericardial effusion	Right bundle branch block
Supraventricular tachycardia	Sick sinus syndrome
Trifasicular block	
Ventricular tachycardia	
**Stroke, not specified as hemorrhagic**	
Inclusions	*Exclusions*
Ischemic stroke	Intracerebral hemorrhage
Stroke, NOS	Transient ischemic attack
**Other cardiovascular diseases**	
	Exclusions
	Abdominal aortic aneurysm
	Hypertension
	Peripheral arterial disease
	Primary pulmonary hypertension
	Pulmonary embolism
	Raynaud's disease
	Secondary pulmonary hypertension
	Subarachnoid hemorrhage
	Subdural hematoma
	Venous thromboembolism, excluding PE

Abbreviations - NOS: not otherwise specified, PE: pulmonary embolus

Additional Text e1: Methods used to predict CVD risks in example Hodgkin lymphoma patients

We present predicted risks for a total of ten hypothetical Hodgkin lymphoma patients – one male and one female patient diagnosed at each of ages 20, 30, 40, 50, and 60. We assume that each of the ten patients has received the same anthracycline-based chemotherapy and the same radiotherapy doses to the organs at risk, and that these were the anthracycline dose and mean radiotherapy doses used to treat an average PET-negative patient with mediastinal Hodgkin lymphoma, based on treatments delivered to the RAPID trial cohort [1,2]. These average radiotherapy doses to the cardiovascular organs at risk are shown in Table 2.

For each patient, cumulative risks for each year up to 30 years following treatment were calculated for incidence of the five relevant CVD categories, based on incidence rates calculated in this study using Method A1. We took account of competing risks due to non-CVD mortality and incidence of CVDs at prior ages, as follows:

Background mortality

It was assumed that, for each patient, their mortality rate in the absence of any treatment effects would be equal to the appropriate age-and-sex-specific mortality rate in the general population. UK mortality rates were extracted from the WHO mortality database for 2013 [3] in 5-year age groups for male and female patients. Rates of CVD mortality were subtracted from all-cause mortality [2]. The predicted 30-year background CVD incidence risk for each patient, taking into account competing risks of non-CVD mortality or prior incident CVD ("competing risks"), was then calculated as follows.

Calculation of predicted CVD incidence taking account of competing risks

Let the annual incidence rate for the specific disease of interest during the nth year after treatment be denoted by r_n^{sp}, and the corresponding death rate for all non-CVD causes be denoted by r_n^{oth}. Then the probability — or risk — of incidence of the specific disease of interest during year n, given that the individual has survived up to the beginning of year n and not experienced a CVD, is:

$$\boldsymbol{p_n^{sp} = 1 - e^{-r_n^{sp}}} \ .$$

Also, the probability that the individual experienced the specific disease of interest or died of a non-CVD cause, given survival up to the beginning of year n and no incident CVD, is:

$$\boldsymbol{p_n^{sp+oth} = 1 - e^{-(r_n^{sp} + r_n^{oth})}} \ ,$$

and the probability that the individual survived without experiencing a CVD to the end of year n, given survival up to the beginning of year n, is:

$$\boldsymbol{q_n^{sp+oth} = 1 - p_n^{sp+oth}} \ .$$

Hence, the cumulative probability that the individual survived up to the beginning of year n and experienced no CVD is:

$$\boldsymbol{Q_{n-1}^{sp+oth} = Q_{n-2}^{sp+oth} \times q_{n-1}^{sp+oth}} \ ,$$

where $Q_0^{sp+oth} = 1$,
i.e. the individual is assumed to be alive at the start and CVD-naive,

and the probability that the individual survived up to the beginning of year n and experienced a CVD during year n:

$$\boldsymbol{p\_cr_n^{sp}} = \boldsymbol{p_n^{sp}} \times \boldsymbol{Q_{n-1}^{sp+oth}} \quad .$$

The cumulative probability —or cumulative risk — that the individual has experienced incident CVD by the end of year n (n-year cumulative incidence) is therefore:

$$\sum_{i=1}^{n} \boldsymbol{p\_cr_i^{sp}}$$

Predicting incidence risks with treatments for Hodgkin Lymphoma

Predictions of the 30-year cumulative incidence risk for each of the five CVD categories, taking into account the likely effects of treatment, were then obtained in a similar fashion to that described above for the prediction of 30-year cumulative background mortality and incidence risks. The effects of treatment were based on relative rates of CVD incidence derived from prior publications (Table 1). These were multiplied by age- and sex-specific CVD rates to obtain rates expected after treatment. Steps to take account of competing risks were then taken, as described above.

Figure 6 in the main paper shows the predicted cumulative risks for four of the example Hodgkin lymphoma patients if they received no treatment (black lines), if they received anthracycline-based chemotherapy only (blue lines), and if they received both anthracycline-based chemotherapy and radiotherapy (orange lines). Table 3 gives 30-year predicted risks with and without treatment for all ten example patients. Full details of the methods used to calculate cumulative risks, competing risks, and predicted excess risks due to anthracycline-based chemotherapy and radiotherapy have previously been published [2].

Additional Results: Figures

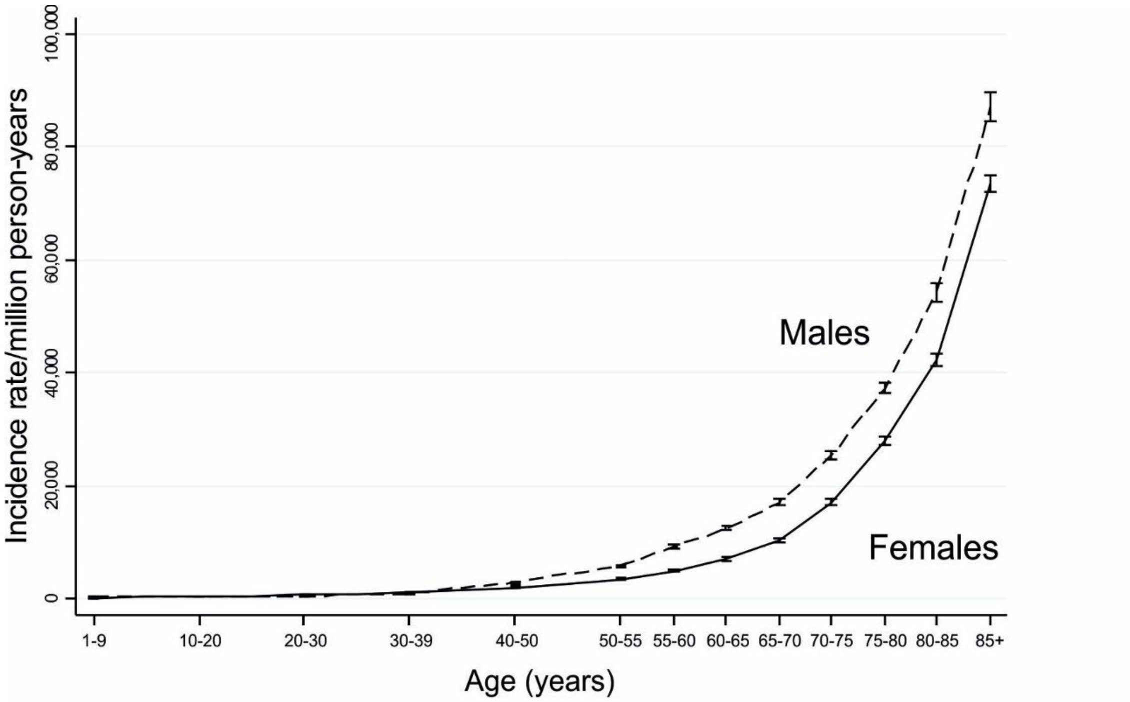

Additional Figure e1. Incidence rates of all relevant CVDs, by age and sex, and including conduction defects and pericardial effusion (Method A1).

Incidence rate of all relevant CVDs: Males

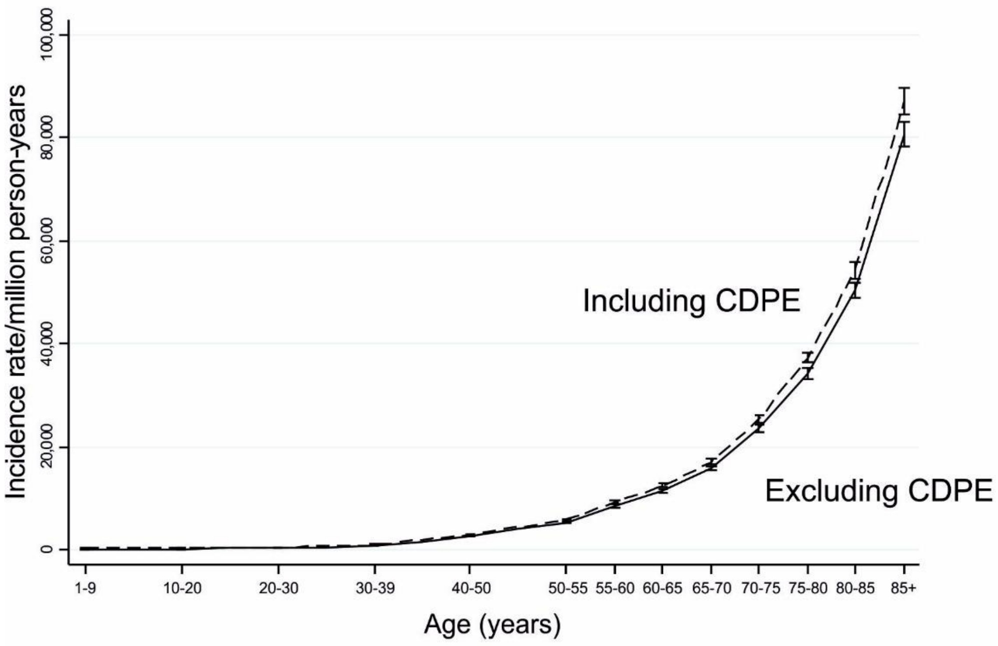

Incidence rate of all relevant CVDs: Females

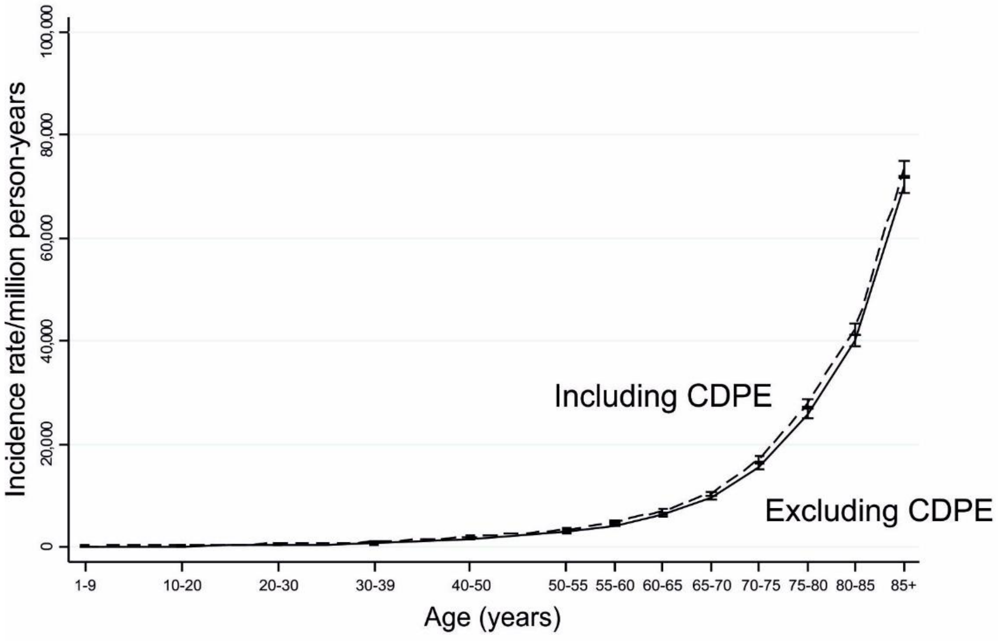

Additional Figure e2. Incidence rates for all relevant cardiovascular diseases, including and excluding events recorded as conduction defects and pericardial effusion, by age. Top panel displays rates for females. Bottom panel displays rates for males.

All relevant CVDs, ignoring conduction defects and pericardial effusion (Method A2)

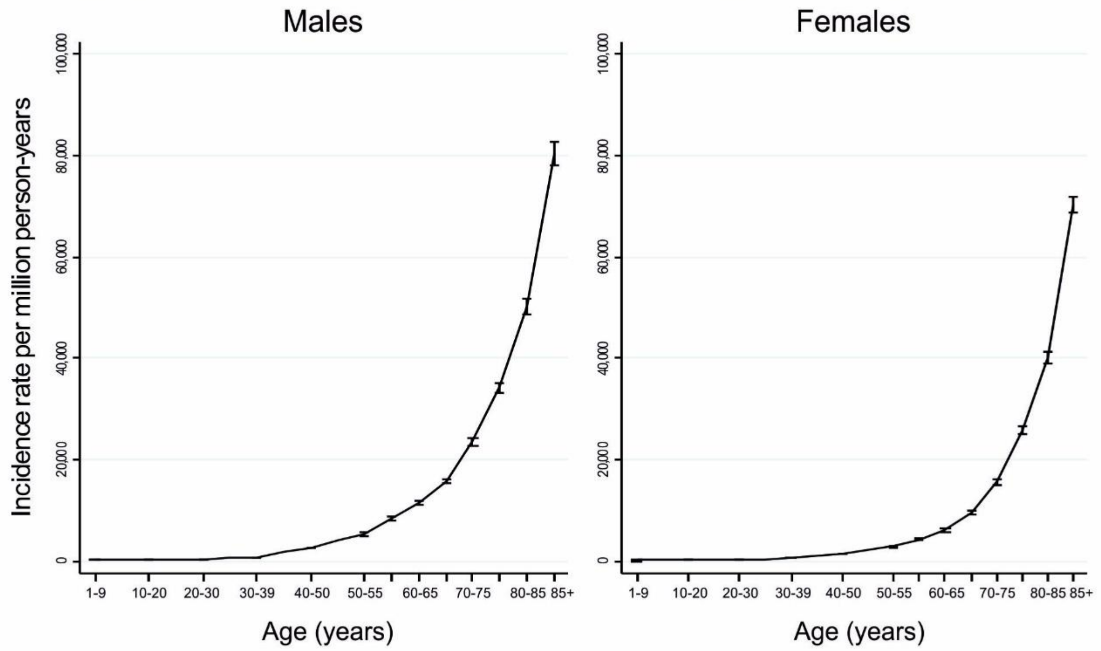

Contributions of individual CVD categories to all relevant CVDs, ignoring conduction defects and pericardial effusion (Method A2)

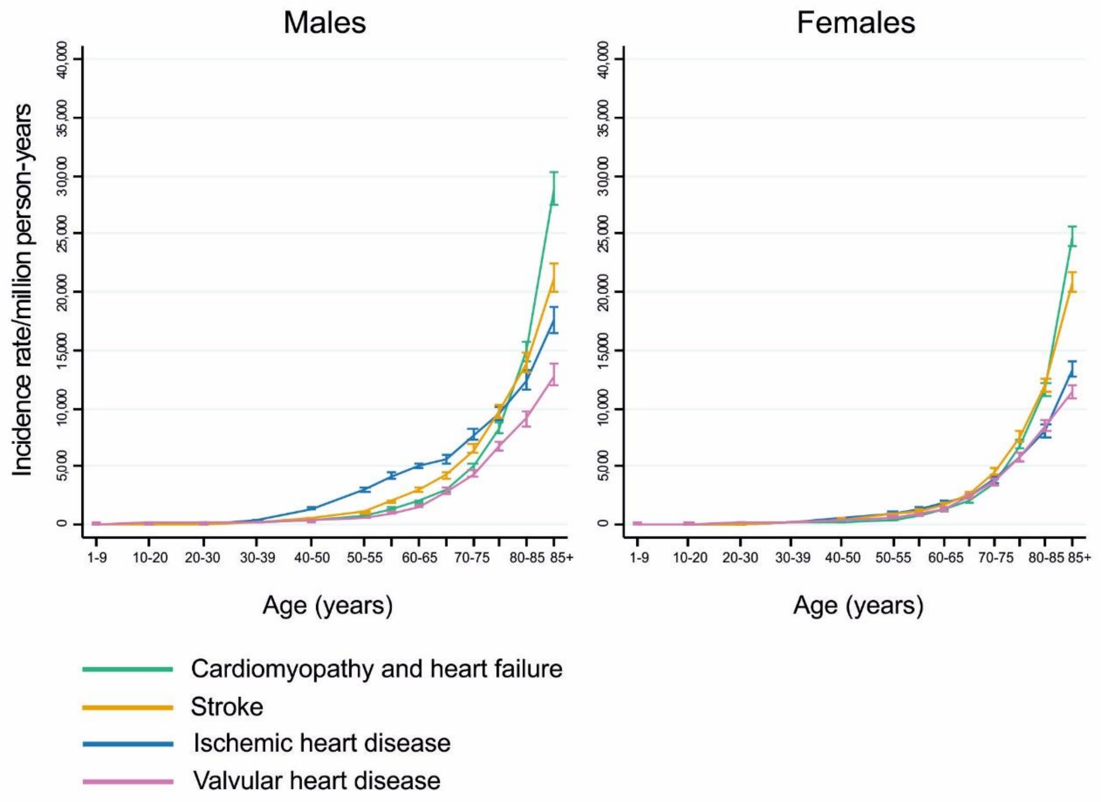

Additional figure e3: Incidence rate of all relevant CVDs, ignoring conduction defects and pericardial effusion, by age and sex (Method A2). Top row: incidence rate for all relevant CVDs combined, excluding conduction defects and pericardial effusion, and calculated using Method A2. Bottom row: the contributions of individual CVD categories to the incidence rate of all relevant CVDs combined. In all four panels only an individual's first recorded diagnosis of any relevant CVD is included (see Tables 1 and e3). For each age and sex group, the sum of the rates across the four categories in the bottom row is equal to the corresponding rate for all relevant CVDs in the top row.

All relevant heart diseases, ignoring conduction defects and pericardial effusion (Method B2)

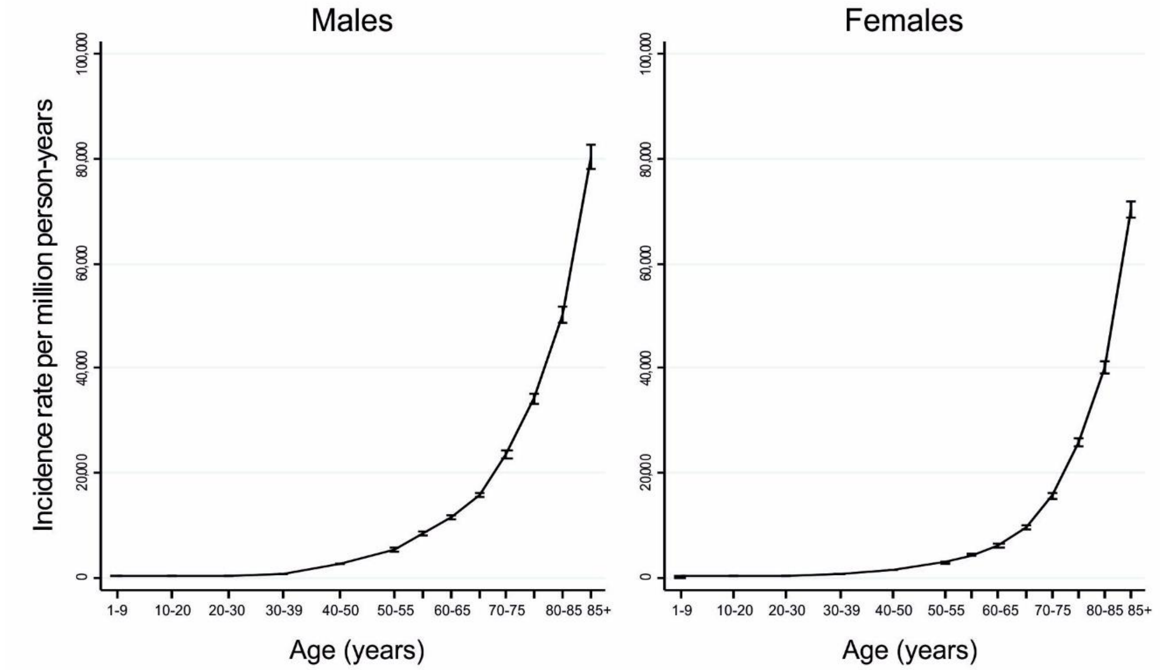

Contributions of individual CVD categories to all relevant heart diseases, ignoring conduction defects and pericardial effusion (Method B2)

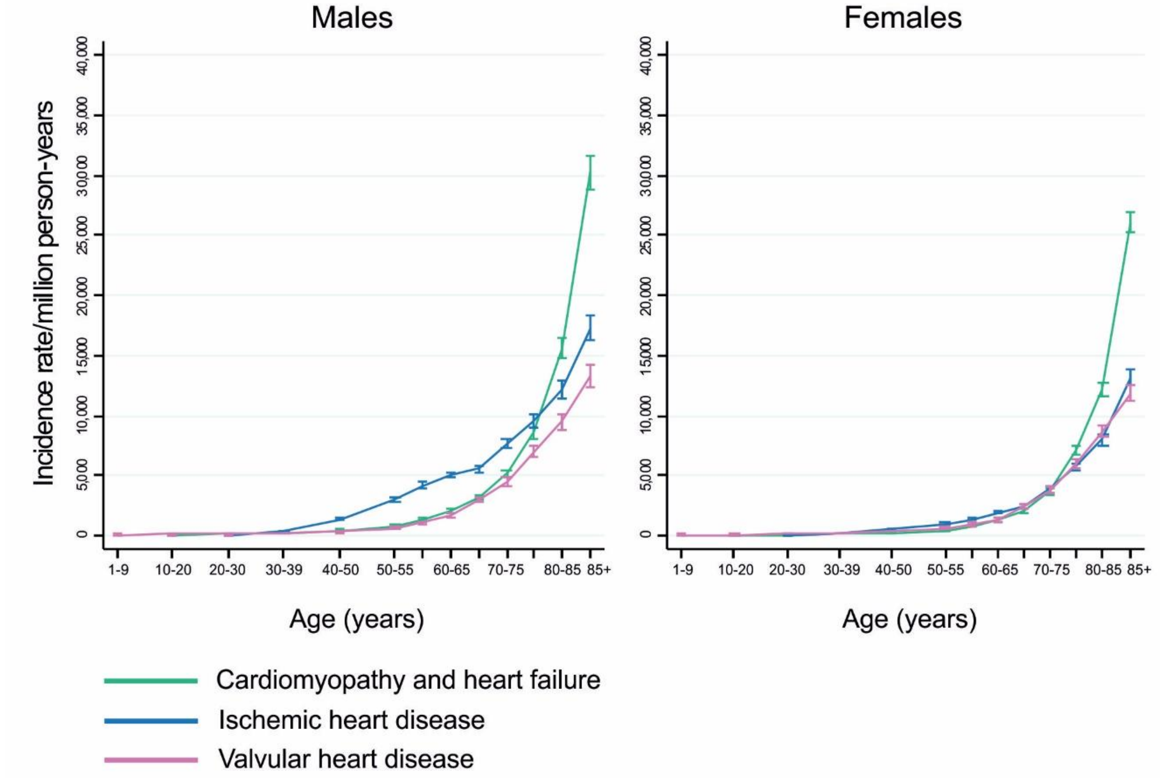

Additional figure e4: Incidence rate of all relevant heart diseases, ignoring conduction defects and pericardial effusion, by age and sex (Method B2). Top row: incidence rate for all relevant heart diseases combined, excluding conduction defects and pericardial effusion, and calculated using Method B2. Bottom row: the contributions of individual heart disease categories to the incidence rate of all relevant heart diseases combined. In all four panels only an individual's first recorded diagnosis of any relevant heart disease is included (see Tables 1 and e5). For each age and sex group, the sum of the rates across the three categories in the bottom row is equal to the corresponding rate for all relevant heart diseases in the top row.

Additional Results: Tables

Additional Table e2: Contributions of each of the five categories to incidence rates of all relevant CVDs (Method A1)

For each individual only the first recorded diagnosis of CVD or of heart disease is included (i.e. method A1 in Figure 2).

Age (yrs)	Ischemic heart disease	Cardiomyopathy & heart failure	Valvular heart disease	Conduction defects & pericardial effusion	Non-hemorrhagic stroke
**Females**	Rate* *95% CI*	Rate* *95% CI*	Rate* *95% CI*	Rate* *95% CI*	Rate* *95% CI*
1-9	-- -	-- -	82 *58 - 116*	59 *39 - 88*	-- -
10-19	-- -	30 *19 - 48*	79 *59 - 105*	182 *151 - 220*	25 *15 - 41*
20-29	31 *19 - 51*	65 *46 - 92*	145 *115 - 183*	314 *268 - 368*	66 *47 - 93*
30-39	115 *91-146*	112 *89 - 143*	196 *164 - 235*	353 *308 - 403*	185 *153 - 222*
40-49	481 *436 - 531*	223 *193 - 257*	335 *298 - 377*	488 *442 - 538*	425 *383 - 472*
50-54	967 *873 - 1070*	426 *365 - 496*	544 *475 - 623*	561 *491 - 641*	855 *768 - 953*
55-59	1357 *1238 - 1488*	766 *678 - 866*	860 *767 - 966*	804 *713 - 906*	1142 *1033 - 1263*
60-64	1923 *1779 - 2078*	1188 *1076 - 1311*	1244 *1129 - 1370*	927 *829 - 1037*	1717 *1581 - 1864*
65-69	2488 *2314 - 2674*	1931 *1779 - 2096*	2287 *2121 - 2466*	1134 *1019 - 1262*	2586 *2409 - 2776*
70-74	3847 *3596 - 4115*	3479 *3241 - 3735*	3595 *3353 - 3855*	1550 *1394 - 1724*	4504 *4231 - 4793*
75-79	5754 *5411 - 6118*	6641 *6272 - 7031*	5655 *5315 - 6016*	2138 *1933 - 2365*	7584 *7189 - 8001*
80-84	8074 *7599 - 8579*	11,471 *10,902 - 12,069*	8271 *7790 - 8781*	2551 *2290 - 2842*	11,972 *11,391 - 12,583*
85+	13,384 *12,746 - 14,054*	24,323 *23,457 - 25,220*	11,055 *10,476 - 11,665*	3816 *3482 - 4181*	20,914 *20,112 - 21,747*
No. cases	7197	8036	6317	3532	8956
Person yrs	4,892,483	4,892,483	4,892,483	4,892,483	4,892,483
Crude rate	1471 *1437 - 1505*	1643 *1607 - 1679*	1291 *1260 - 1323*	722 *698 - 746*	1830 *1793 - 1869*
Age-std	1454 *1421 - 1488*	1650 *1614 - 1686*	1292 *1260 - 1323*	712 *688 - 735*	1828 *1790 - 1865*
**Males**					
1-9	-- -	-- -	72 *50 - 103*	91 *66 - 126*	32 *18 - 54*
10-19	-- -	42 *29 - 62*	85 *65 - 110*	149 *122 - 181*	22 *13 - 36*
20-29	57 *41 - 81*	92 *70 - 121*	97 *74 - 126*	189 *156 - 229*	34 *22 - 53*
30-39	299 *258 - 345*	147 *119 - 180*	132 *106 - 164*	199 *167 - 238*	144 *117 - 178*
40-49	1374 *1296 - 1457*	419 *377 - 466*	269 *236 - 307*	302 *267 - 342*	516 *470 - 568*
50-54	2924 *2758 - 3099*	745 *663 - 836*	536 *468 - 614*	479 *415 - 553*	1089 *989 - 1198*
55-59	4178 *3963 - 4406*	1286 *1169 - 1415*	984 *882 - 1097*	693 *608 - 789*	1972 *1826 - 2131*
60-64	5028 *4782 - 5285*	1885 *1737 - 2044*	1535 *1403 - 1680*	1028 *921 - 1148*	2902 *2717 - 3098*
65-69	5619 *5338 - 5915*	2910 *2709 - 3125*	2810 *2613 - 3021*	1482 *1341 - 1637*	4237 *3994 - 4495*
70-74	7771 *7372 - 8191*	4785 *4475 - 5117*	4247 *3955 - 4560*	2029 *1830 - 2249*	6464 *6102 - 6848*
75-79	9611 *9091 - 10,161*	7986 *7513 - 8488*	6520 *6094 - 6975*	3424 *3120 - 3759*	9696 *9174 - 10,248*
80-84	12,513 *11,765 - 13,308*	14,366 *13,563 - 15,215*	8745 *8124 - 9414*	4632 *4186 - 5125*	13,983 *13,191 - 14,821*
85+	17,790 *16,707 - 18,943*	28,155 *26,783 - 29,596*	12,128 *11,240 - 13,087*	7559 *6865 - 8324*	21,390 *20,199 - 22,651*
No. cases	11,464	7156	5143	3315	8327
Person yrs	4,773,726	4,773,726	4,773,726	4,773,726	4,773,726
Crude	2401 *2358 - 2446*	1499 *1465 - 1534*	1077 *1048 - 1107*	694 *671 - 718*	1744 *1707 - 1782*
Age-std rate	2857 *2805 - 2910*	2127 *2078 - 2177*	1427 *1388 - 1466*	883 *853 - 913*	2309 *2259 - 2358*

Table continued on next page

Table e2 continued

**Both**					
1-9	-- *-*	16 *9 - 28*	77 *60 - 98*	75 *59 - 97*	27 *18 - 42*
10-19	-- *-*	36 *27 - 49*	82 *68 - 99*	165 *144 - 189*	23 *16 - 33*
20-29	45 *34 - 60*	79 *64 - 99*	119 *100 - 142*	247 *219 - 280*	49 *37 - 64*
30-39	207 *183 - 235*	130 *111 - 151*	164 *142 - 188*	275 *247 - 307*	164 *143 - 189*
40-49	928 *883 - 976*	321 *295 - 349*	302 *277 - 330*	395 *366 - 426*	471 *439 - 505*
50-54	1947 *1851 - 2048*	586 *534 - 642*	540 *491 - 595*	520 *471 - 573*	972 *905 - 1044*
55-59	2753 *2629 - 2882*	1023 *949 - 1103*	922 *851 - 998*	749 *686 - 818*	1553 *1461 - 1651*
60-64	3420 *3279 - 3566*	1524 *1431 - 1622*	1384 *1296 - 1478*	976 *902 - 1056*	2288 *2174 - 2409*
65-69	3955 *3792 - 4124*	2389 *2264 - 2522*	2532 *2403 - 2668*	1297 *1205 - 1395*	3360 *3210 - 3516*
70-74	5607 *5379 - 5844*	4065 *3871 - 4268*	3887 *3698 - 4086*	1765 *1639 - 1900*	5383 *5160 - 5616*
75-79	7382 *7083 - 7692*	7208 *6914 - 7516*	6020 *5751 - 6301*	2681 *2504 - 2871*	8475 *8156 - 8808*
80-84	9781 *9368 - 10,213*	12,584 *12,114 - 13,072*	8453 *8070 - 8855*	3351 *3113 - 3608*	12,745 *12,272 - 13,237*
85+	14,762 *14,203 - 15,342*	25,520 *24,783 - 26,280*	11,390 *10,901 - 11,901*	4986 *4666 - 5328*	21,063 *20,394 - 21,754*
No. cases	18,660	15,192	11,460	6847	17,282
Person yrs	9,666,209	9,666,209	9,666,209	9,666,209	9,666,209
Crude rate	1930 *1903 - 1958*	1572 *1547 - 1597*	1186 *1164 - 1207*	708 *692 - 725*	1788 *1761 - 1815*
Age-std rate	2091 *2061 - 2121*	1852 *1823 - 1882*	1347 *1322 - 1372*	767 *749 - 785*	2044 *2013 - 2074*

*Rates are given per 1,000,000 person years. Cases and person years are numbers; all other figures are rates.
Abbreviations – yrs: years; CI: confidence interval; No.cases: total number of cases; Person yrs: Total person-years; Age-std: age-standardized

Additional Table e3: Contributions of each of the four categories to incidence rates of all relevant CVDs, ignoring conduction defects and pericardial effusion (Method A2)

Age (yrs)	Ischemic heart disease		Cardiomyopathy & heart failure		Valvular heart disease		Non-hemorrhagic stroke	
**Females**	Rate*	*95% CI*	Rate*	*95% CI*	Rate*	*95% CI*	Rate*	*95% CI*
1-9	--	-	--	-	82	*58-115*	--	-
10-19	--	-	31	*20-49*	81	*61-107*	25	*15-41*
20-29	31	*19-51*	68	*48-96*	148	*118-187*	66	*47-93*
30-39	115	*91-146*	117	*92-147*	201	*168-240*	185	*153-222*
40-49	481	*436-531*	229	*198-264*	343	*306-386*	425	*383-472*
50-54	966	*873-1069*	444	*382-516*	574	*503-655*	854	*767-952*
55-59	1355	*1236-1486*	784	*695-885*	899	*803-1006*	1141	*1032-1261*
60-64	1920	*1776-2075*	1219	*1106-1344*	1267	*1152-1395*	1714	*1579-1861*
65-69	2483	*2309-2669*	1983	*1829-2151*	2369	*2200-2551*	2581	*2404-2771*
70-74	3838	*3587-4106*	3600	*3358-3860*	3673	*3428-3935*	4493	*4221-4782*
75-79	5735	*5394-6098*	6825	*6451-7220*	5777	*5435-6142*	7560	*7166-7975*
80-84	8039	*7567-8542*	11,613	*11,042-12,214*	8466	*7980-8981*	11,921	*11,342-12,529*
85	13,316	*12,682-13,983*	24,746	*23,875-25,649*	11,388	*10,803-12,006*	20,807	*20,010-21,637*
No. cases	7197		8244		6510		8956	
Person yrs	4,898,474		4,898,474		4,898,474		4,898,474	
Crude rate	1469	*1436-1503*	1683	*1647-1720*	1329	*1297-1362*	1828	*1791-1866*
Age-std rate	1450	*1416-1483*	1686	*1650-1723*	1327	*1294-1359*	1821	*1784-1859*
**Males**								
1-9	--	-	--	-	73	*51-104*	32	*18-54*
10-19	--	-	44	*30-63*	85	*66-111*	22	*13-36*
20-29	57	*41-81*	94	*72-123*	100	*76-129*	34	*22-53*
30-39	299	*258-345*	160	*131-195*	135	*109-168*	144	*117-178*
40-49	1373	*1295-1456*	436	*393-483*	280	*246-319*	516	*469-568*
50-54	2921	*2756-3097*	783	*700-877*	550	*481-630*	1088	*989-1197*
55-59	4174	*3958-4401*	1332	*1213-1463*	1000	*897-1114*	1970	*1824-2128*
60-64	5020	*4776-5276*	1960	*1810-2123*	1576	*1442-1722*	2897	*2713-3093*
65-69	5605	*5325-5900*	3029	*2825-3248*	2885	*2686-3099*	4227	*3985-4484*
70-74	7749	*7351-8168*	4958	*4642-5295*	4379	*4083-4697*	6446	*6084-6829*
75-79	9565	*9047-10,112*	8255	*7776-8765*	6723	*6291-7184*	9649	*9130-10,199*
80-84	12,428	*11,686-13,218*	41942	*14,126-15,805*	9102	*8470-9781*	13,888	*13,103-14,722*
85	17,614	*16,541-18,756*	28,907	*27,523-30,359*	12,795	*11,886-13,773*	21,178	*19,999-22,427*
No. cases	11,464		7453		5336		8327	
Person yrs	4,778,987		4,778,987		4,778,987		4,778,987	
Crude rate	2399	*2355-2443*	1560	*1525-1595*	1117	*1087-1147*	1742	*1705-1780*
Age-std rate	2846	*2794-2898*	2201	*2151-2251*	1478	*1439-1518*	2297	*2248-2346*

Table continued on next page

Table e3 continued

**Both**				
1-9	-- -	16 *9-28*	77 *60-99*	27 *18-42*
10-19	-- -	38 *28-50*	83 *69-101*	23 *16-33*
20-29	45 *34-60*	82 *66-101*	122 *103-145*	49 *37-64*
30-39	207 *183-234*	138 *119-161*	168 *147-193*	164 *143-189*
40-49	927 *882-975*	332 *306-361*	312 *286-340*	471 *439-505*
50-54	1945 *1849-2046*	614 *561-672*	562 *512-618*	971 *904-1043*
55-59	2749 *2626-2878*	1055 *980-1136*	949 *877-1026*	1551 *1459-1649*
60-64	3414 *3274-3561*	1576 *1482-1677*	1416 *1327-1511*	2284 *2170-2405*
65-69	3946 *3784-4114*	2473 *2346-2608*	2611 *2480-2749*	3352 *3203-3508*
70-74	5592 *5365-5829*	4209 *4013-4416*	3990 *3798-4191*	5369 *5146-5601*
75-79	7353 *7056-7662*	7429 *7131-7740*	6177 *5905-6461*	8442 *8124-8773*
80-84	9730 *9318-10,159*	12,895 *12,420-13,388*	8711 *8322-9118*	12,679 *12,208-13,167*
85+	14,664 *14,109-15,241*	26,051 *25,308-26,816*	11,829 *11,332-12,348*	20,924 *20,259-21,610*
No. cases	18,660	15,697	11,846	17,282
Person yrs	9,677,461	9,677,461	9,677,461	9,677,461
Crude rate	1928 *1901-1956*	1622 *1597-1648*	1224 *1202-1246*	1786 *1759-1813*
Age-std rate	2084 *2054-2114*	1904 *1875-1934*	1388 *1363-1413*	2036 *2005-2066*

*Rates are given per 1,000,000 person years. Cases and person years are numbers; all other figures are rates.
Abbreviations – yrs: years; CI: confidence interval; No.cases: total number of cases; Person yrs: Total person-years; Age-std: age-standardized

Additional Table e4: Contributions of each of the four categories to incidence rates of all relevant heart diseases (Method B1)

Age (yrs)	Ischemic heart disease		Cardiomyopathy & heart failure		Valvular heart disease		Conduction defects & pericardial effusion	
**Females**	Rate*	*95% CI*	Rate*	*95% CI*	Rate*	*95% CI*	Rate*	*95% CI*
1-9	--	-	--	-	82	*58-116*	59	*39-88*
10-19	--	-	30	*19-48*	79	*59-105*	182	*151-220*
20-29	31	*19-51*	65	*46-92*	147	*117-186*	314	*268-368*
30-39	116	*92-147*	112	*89-143*	199	*167-238*	354	*310-405*
40-49	482	*437-532*	229	*198-264*	340	*302-382*	491	*445-541*
50-54	971	*877-1074*	438	*377-510*	572	*501-653*	568	*498-648*
55-59	1359	*1240-1489*	790	*701-891*	877	*782-983*	811	*720-913*
60-64	1925	*1781-2080*	1242	*1128-1368*	1274	*1158-1402*	931	*832-1041*
65-69	2498	*2325-2685*	1969	*1816-2136*	2344	*2176-2525*	1132	*1017-1260*
70-74	3861	*3611-4129*	3577	*3336-3835*	3689	*3444-3951*	1608	*1449-1784*
75-79	5736	*5395-6098*	6881	*6507-7276*	5825	*5482-6190*	2189	*1983-2417*
80-84	7992	*7523-8489*	12,018	*11,440-12,624*	8467	*7984-8978*	2611	*2349-2902*
85+	13,241	*12,614-13,899*	25,682	*24,803-26,592*	11,460	*10,878-12,073*	3881	*3549-4245*
No. cases	7259		8513		6570		3607	
Person yrs	4,905,948		4,905,948		4,905,948		4,905,948	
Crude	1480	*1446-1514*	1735	*1699-1772*	1339	*1307-1372*	735	*712-760*
Age-std	1450	*1417-1483*	1721	*1684-1757*	1327	*1295-1360*	721	*698-745*
**Males**								
1-9	--	-	--	-	72	*50-103*	91	*66-126*
10-19	--	-	42	*29-62*	85	*65-110*	149	*122-181*
20-29	57	*41-81*	92	*70-121*	99	*76-129*	189	*156-229*
30-39	300	*260-347*	151	*123-185*	136	*110-169*	199	*167-238*
40-49	1375	*1297-1457*	432	*389-479*	281	*247-319*	305	*269-345*
50-54	2921	*2755-3096*	751	*669-843*	566	*496-646*	486	*421-561*
55-59	4172	*3957-4399*	1308	*1190-1437*	1008	*905-1123*	699	*615-796*
60-64	5031	*4787-5287*	1948	*1798-2109*	1584	*1449-1730*	1034	*926-1153*
65-69	5597	*5317-5891*	3019	*2815-3237*	2917	*2717-3131*	1515	*1373-1672*
70-74	7716	*7321-8133*	4976	*4661-5312*	4368	*4074-4684*	2089	*1888-2311*
75-79	9574	*9059-10,118*	8259	*7782-8766*	6724	*6295-7183*	3511	*3205-3847*
80-84	12,307	*11,574-13,086*	14,960	*14,149-15,816*	9086	*8460-9759*	4847	*4395-5345*
85+	17,466	*16,407-18,593*	29,521	*28,136-30,976*	12,633	*11,738-13,597*	7785	*7089-8550*
No. cases	11,519		7530		5390		3423	
Person yrs	4,787,358		4,787,358		4,787,358		4,787,358	
Crude	2406	*2363-2450*	1573	*1538-1609*	1126	*1096-1156*	715	*691-739*
Age-std	2839	*2787-2890*	2211	*2161-2261*	1477	*1438-1517*	904	*873-934*

Table continued on next page

Table e4 continued

**Both**				
1-9	-- -	17 *10-29*	77 *60-98*	75 *59-97*
10-19	-- -	36 *27-49*	82 *68-99*	165 *144-189*
20-29	45 *34-60*	79 *64-99*	121 *102-144*	247 *219-280*
30-39	208 *184-236*	132 *113-154*	167 *146-192*	276 *248-307*
40-49	929 *884-977*	330 *304-359*	310 *284-338*	398 *369-430*
50-54	1947 *1851-2048*	595 *543-652*	569 *518-625*	527 *478-581*
55-59	2751 *2628-2880*	1046 *971-1127*	942 *871-1018*	756 *692-825*
60-64	3424 *3283-3570*	1583 *1488-1683*	1423 *1334-1519*	980 *907-1060*
65-69	3952 *3790-4120*	2462 *2335-2595*	2613 *2482-2750*	1312 *1220-1411*
70-74	5593 *5367-5830*	4205 *4009-4411*	3994 *3804-4195*	1824 *1696-1961*
75-79	7360 *7064-7668*	7464 *7166-7774*	6206 *5934-6489*	2749 *2570-2939*
80-84	9656 *9249-10,081*	13,152 *12,676-13,647*	8706 *8320-9109*	3473 *3233-3732*
85+	14,565 *14,017-15,134*	26,885 *26,137-27,654*	11,827 *11,335-12,341*	5105 *4785-5446*
No. cases	18,778	16,043	11,960	7030
Person yrs	9,693,306	9,693,306	9,693,306	9,693,306
Crude	1937 *1910-1965*	1655 *1630-1681*	1234 *1212-1256*	725 *708-742*
Age-std	2082 *2052-2111*	1929 *1899-1959*	1389 *1364-1414*	781 *763-800*

*Rates are given per 1,000,000 person years. Cases and person years are numbers; all other figures are rates.
Abbreviations – yrs: years; CI: confidence interval; No.cases: total number of cases; Person yrs: Total person-years; Age-std: age-standardized

Additional Table e5: Contributions of each of the three categories to incidence rates of all relevant heart diseases, ignoring CDPE (Method B2)

Age (yrs)	Ischemic heart disease		Cardiomyopathy & heart failure		Valvular heart disease	
**Females**	Rate*	*95% CI*	Rate*	*95% CI*	Rate*	*95% CI*
1-9	--	-	-	-	82	*58-115*
10-19	--	-	31	*20-49*	81	*61-107*
20-29	31	*19-51*	68	*48-96*	151	*120-189*
30-39	116	*92-146*	117	*92-147*	204	*171-244*
40-49	482	*437-532*	235	*204-270*	348	*310-391*
50-54	970	*876-1073*	456	*394-529*	604	*531-687*
55-59	1357	*1238-1487*	809	*719-911*	915	*818-1023*
60-64	1922	*1778-2077*	1274	*1158-1401*	1301	*1184-1429*
65-69	2493	*2320-2679*	2022	*1866-2190*	2426	*2255-2610*
70-74	3852	*3602-4119*	3697	*3452-3959*	3771	*3524-4036*
75-79	5717	*5378-6077*	7075	*6696-7475*	5953	*5607-6321*
80-84	7957	*7491-8452*	12,186	*11,605-12,795*	8664	*8177-9180*
85+	13,172	*12,548-13,827*	26,094	*25,210-27,009*	11,833	*11,243-12,454*
No. cases	7259		8729		6775	
Person yrs	4,912,064		4,912,064		4,912,064	
Crude	1478	*1444-1512*	1777	*1740-1815*	1379	*1347-1412*
Age-std	1445	*1412-1479*	1758	*1721-1795*	1364	*1332-1397*
**Males**						
1-9	--	-	--	-	73	*51-104*
10-19	--	-	44	*30-63*	85	*66-111*
20-29	57	*41-81*	94	*72-123*	101	*78-131*
30-39	300	*260-347*	164	*135-199*	139	*113-172*
40-49	1374	*1296-1456*	450	*407-499*	291	*257-331*
50-54	2918	*2753-3094*	790	*706-883*	580	*509-661*
55-59	4167	*3952-4394*	1353	*1233-1485*	1024	*920-1139*
60-64	5022	*4779-5278*	2023	*1871-2188*	1625	*1489-1773*
65-69	5583	*5304-5876*	3141	*2934-3363*	2993	*2790-3209*
70-74	7693	*7299-8108*	5146	*4826-5488*	4499	*4200-4819*
75-79	9527	*9015-10,069*	8533	*8049-9046*	6937	*6502-7402*
80-84	12,219	*11,491-12,993*	15,537	*14,713-16,406*	9457	*8820-10,141*
85+	17,282	*16,234-18,397*	30,265	*28,869-31,729*	13,282	*12,368-14,264*
No. cases	11,519		7837		5590	
Person yrs	4,792,811		4,792,811		4,792,811	
Crude	2403	*2360-2448*	1635	*1599-1672*	1166	*1136-1197*
Age-std	2827	*2775-2879*	2285	*2234-2336*	1529	*1489-1569*

Table continued on next page

Table e5 continued

**Both**			
1-9	-- -	17 *10-29*	77 *60-99*
10-19	-- -	38 *28-50*	83 *69-101*
20-29	45 *34-60*	82 *66-101*	124 *105-148*
30-39	208 *184-236*	140 *121-163*	172 *150-197*
40-49	928 *883-976*	343 *316-372*	320 *293-348*
50-54	1946 *1850-2046*	623 *570-682*	592 *540-649*
55-59	2748 *2624-2877*	1078 *1002-1160*	969 *897-1046*
60-64	3418 *3278-3564*	1636 *1539-1738*	1457 *1367-1554*
65-69	3943 *3782-4111*	2547 *2418-2683*	2692 *2559-2831*
70-74	5578 *5352-5814*	4348 *4149-4557*	4098 *3905-4301*
75-79	7330 *7036-7637*	7692 *7390-8006*	6370 *6096-6657*
80-84	9603 *9199-10,026*	13,480 *12,999-13,979*	8970 *8580-9379*
85	14,465 *13,921-15,030*	27,406 *26,653-28,179*	12,289 *11,788-12,810*
No. cases	18,778	16,566	12,364
Person yrs	9,704,874	9,704,874	9,704,874
Crude	1935 *1907-1963*	1707 *1681-1733*	1274 *1252-1297*
Age-std	2074 *2045-2104*	1982 *1952-2012*	1431 *1406-1456*

*Rates are given per 1,000,000 person years. Cases and person years are numbers; all other figures are rates.
Abbreviations – yrs: years; CI: confidence interval; No.cases: total number of cases; Person yrs: Total person-years; Age-std: age-standardized

Additional Table e6: Incidence rates for each of the five cardiovascular disease categories, calculated separately (Method C)

Age (yrs)	Ischemic heart disease	Cardiomyopathy & heart failure	Valvular heart disease	Conduction defects & pericardial effusion	Non-hemorrhagic stroke
**Females**	Rate* *95% CI*	Rate* *95% CI*	Rate* *95% CI*	Rate* *95% CI*	Rate* *95% CI*
1-9	-- -	-- -	82 *58-115*	61 *41-91*	-- -
10-19	-- -	32 *20-50*	83 *63-110*	186 *155-224*	25 *15-41*
20-29	37 *23-59*	76 *55-105*	159 *127-198*	328 *281-383*	66 *47-93*
30-39	129 *103-161*	137 *111-170*	228 *193-270*	377 *331-430*	189 *157-227*
40-49	512 *465-563*	312 *276-353*	410 *368-456*	530 *483-582*	441 *398-489*
50-54	1030 *934-1136*	626 *552-710*	698 *620-787*	667 *591-754*	885 *796-984*
55-59	1463 *1339-1598*	1082 *976-1199*	1106 *999-1224*	928 *830-1036*	1180 *1070-1302*
60-64	2073 *1924-2233*	1702 *1568-1848*	1598 *1468-1739*	1096 *989-1214*	1793 *1655-1943*
65-69	2658 *2480-2849*	2696 *2517-2889*	2967 *2778-3169*	1411 *1283-1552*	2735 *2554-2929*
70-74	4319 *4055-4600*	5051 *4765-5354*	4711 *4436-5004*	2160 *1976-2361*	4730 *4453-5024*
75-79	6603 *6241-6986*	9350 *8917-9804*	7807 *7412-8223*	2882 *2647-3138*	7998 *7598-8419*
80-84	9425 *8924-9954*	15,993 *15,333-16,680*	11,690 *11,129-12,279*	3596 *3293-3928*	12,664 *12,079-13,277*
85+	15,552 *14,889-16,245*	33,197 *32,213-24,211*	17,283 *16,581-18,015*	5311 *4932-5720*	22,669 *21,862-23,506*
No. cases	8387	11,637	9000	4530	9854
Person yrs	4,935,493	4,933,281	4,933,277	4,940,722	4,933,254
Crude	1699 *1663-1736*	2359 *2316-2402*	1824 *1787-1862*	917 *891-944*	1997 *1958-2037*
Age-std	1637 *1601-1672*	2297 *2255-2339*	1777 *1740-1814*	888 *862-914*	1937 *1898-1975*
**Males**					
1-9	-- -	-- -	73 *51-104*	92 *67-127*	32 *18-54*
10-19	-- -	45 *31-64*	89 *69-115*	154 *127-187*	23 *14-38*
20-29	57 *41-81*	111 *87-143*	113 *88-145*	192 *159-232*	34 *22-53*
30-39	308 *267-355*	208 *175-248*	166 *136-201*	223 *188-264*	147 *120-181*
40-49	1409 *1330-1492*	649 *597-707*	407 *366-453*	413 *372-459*	540 *492-593*
50-54	3025 *2857-3203*	1301 *1192-1419*	809 *725-904*	686 *608-773*	1118 *1018-1228*
55-59	4341 *4122-4572*	2099 *1948-2260*	1414 *1291-1547*	992 *890-1105*	2056 *1908-2216*
60-64	5329 *5078-5591*	3044 *2857-3244*	2327 *2164-2503*	1485 *1356-1626*	3036 *2849-3236*
65-69	6020 *5732-6323*	4613 *4362-4878*	4006 *3772-4253*	2062 *1896-2241*	4431 *4185-4691*
70-74	8448 *8038-8879*	7315 *6935-7716*	6090 *5744-6456*	2981 *2743-3240*	6875 *6507-7264*
75-79	10,722 *10,184-11,288*	11,949 *11,380-12,545*	9547 *9040-10,082*	4823 *4469-5206*	10,356 *9828-10,913*
80-84	14,152 *13,380-14,968*	21,068 *20,120-22,061*	13,726 *12,966-14,529*	6550 *6034-7109*	14,854 *14,062-15,691*
85+	20,722 *19,603-21,905*	39,539 *37,967-41,175*	20,239 *19,132-21,411*	10,797 *10,001-11,655*	22,578 *21,405-23,815*
No. cases	12,719	11,259	7984	4776	9126
Person yrs	4,813,881	4,819,517	4,822,209	4,827,630	4,820,239
Crude	2642 *2597-2688*	2336 *2293-2380*	1656 *1620-1692*	989 *962-1018*	1893 *1855-1933*
Age-std	3114 *3060-3168*	3162 *3104-3221*	2154 *2107-2202*	1228 *1193-1263*	2437 *2387-2487*

Table continued on next page

Table e6 continued

**Both**					
1-9	-- *-*	17 *10-29*	77 *60-99*	77 *60-99*	27 *18-42*
10-19	-- *-*	38 *29-51*	86 *72-104*	170 *148-194*	24 *17-34*
20-29	48 *36-63*	95 *78-116*	134 *114-158*	255 *226-288*	49 *37-64*
30-39	219 *194-247*	173 *151-198*	197 *173-223*	300 *270-332*	168 *146-193*
40-49	961 *914-1009*	481 *449-516*	408 *379-440*	471 *439-506*	491 *458-526*
50-54	2029 *1931-2132*	964 *898-1036*	754 *695-818*	677 *621-737*	1002 *934-1075*
55-59	2888 *2762-3020*	1586 *1494-1685*	1259 *1177-1347*	960 *888-1037*	1615 *1521-1714*
60-64	3646 *3501-3796*	2352 *2236-2473*	1951 *1846-2062*	1285 *1200-1375*	2395 *2278-2517*
65-69	4238 *4071-4411*	3598 *3445-3758*	3457 *3307-3613*	1718 *1613-1829*	3534 *3382-3692*
70-74	6179 *5942-6425*	6073 *5838-6316*	5334 *5114-5563*	2531 *2382-2690*	5698 *5471-5934*
75-79	8352 *8041-8676*	10,456 *10,106-10,817*	8548 *8233-8875*	3708 *3504-3925*	9001 *8677-9337*
80-84	11,256 *10,824-11,705*	17,962 *17,412-18,529*	12,481 *12,026-12,955*	4743 *4467-5036*	13,514 *13,038-14,006*
85+	17,186 *16,607-17,786*	35,200 *34,360-36,061*	18,220 *17,622-18,838*	7044 *6679-7429*	22,640 *21,972-23,329*
No. cases	21,106	22,896	16,984	9306	18,980
Person yrs	9,749,375	9,752,798	9,755,487	9,768,351	9,753,493
Crude	2165 *2136-2194*	2348 *2317-2378*	1741 *1715-1767*	953 *934-972*	1946 *1918-1974*
Age-std	2306 *2275-2337*	2669 *2634-2703*	1935 *1906-1964*	1015 *994-1035*	2165 *2134-2195*

*Rates are given per 1,000,000 person years. Cases and person years are numbers; all other figures are rates.
Abbreviations – yrs: years; CI: confidence interval; No.cases: total number of cases; Person yrs: Total person-years; Age-std: age-standardized

Additional Table e7: Incidence rates of 38 individual cardiovascular diseases, by age and sex

**Age (yrs)**	**Stable angina**		**Myocardial infarction**		**Unstable angina**		**Coronary heart disease, not otherwise specified**	
**Females**	Rate*	*95% CI*	Rate*	*95% CI*	Rate*	*95% CI*	Rate*	*95% CI*
1-9	-	-	--	-	--	-	--	-
10-19	22	*13-37*	--	-	--	-	--	-
20-29	45	*30-69*	27	*15-46*	--	-	27	*15-46*
30-39	144	*117-178*	100	*78-129*	80	*61-106*	62	*45-86*
40-49	695	*641-754*	374	*335-418*	307	*272-347*	206	*178-239*
50-54	1742	*1616-1878*	756	*675-847*	604	*532-685*	504	*438-579*
55-59	2191	*2040-2354*	1207	*1096-1328*	791	*702-890*	765	*678-863*
60-64	3228	*3042-3425*	1725	*1592-1869*	1135	*1029-1253*	1183	*1074-1303*
65-69	4212	*3987-4449*	2299	*2137-2472*	1495	*1367-1636*	1699	*1561-1848*
70-74	5912	*5605-6235*	4099	*3853-4362*	2263	*2083-2459*	2533	*2342-2739*
75-79	7546	*7165-7946*	6596	*6255-6955*	3194	*2961-3445*	3573	*3328-3836*
80-84	8267	*7816-8745*	9064	*8613-9538*	4645	*4329-4983*	4819	*4500-5161*
85	9106	*8639-9598*	15,081	*14,506-15,679*	6353	*5990-6737*	6557	*6191-6945*
No. cases	9893		8817		4813		5019	
Person yrs	5,013,521		5,112,049		5,129,844		5,143,172	
Crude	1973	*1935-2013*	1725	*1689-1761*	938	*912-965*	976	*949-1003*
Age-std	1846	*1810-1882*	1504	*1472-1535*	816	*793-839*	846	*823-869*
**Males**								
1-9	--	-	--	-	--	-	--	-
10-19	--	-	--	-	--	-	--	-
20-29	62	*45-87*	41	*27-62*	25	*15-42*	37	*24-57*
30-39	196	*164-234*	251	*214-294*	150	*122-184*	90	*69-117*
40-49	1214	*1141-1291*	1198	*1126-1275*	688	*634-747*	453	*409-501*
50-54	2807	*2646-2977*	2570	*2417-2733*	1424	*1312-1546*	922	*833-1021*
55-59	4633	*4409-4868*	3867	*3664-4082*	2075	*1928-2232*	1480	*1358-1613*
60-64	6059	*5795-6335*	4779	*4548-5021*	2569	*2403-2746*	2120	*1971-2281*
65-69	7278	*6966-7604*	5890	*5615-6177*	2861	*2675-3060*	2660	*2482-2850*
70-74	10,148	*9708-10,608*	8322	*7938-8725*	4088	*3828-4366*	3843	*3594-4109*
75-79	11,868	*11,321-12,441*	10,650	*10,156-11,168*	5320	*4986-5677*	4883	*4568-5220*
80-84	11,926	*11,255-12,636*	14,155	*13,463-14,883*	6682	*6231-7166*	6339	*5908-6801*
85	12,727	*11,927-13,581*	21,270	*20,280-22,308*	8493	*7906-9124*	8193	*7627-8801*
No. cases	13,518		13,411		7011		6121	
Person yrs	4,900,102		4,977,009		5,045,684		5,082,397	
Crude	2759	*2713-2806*	2695	*2649-2741*	1390	*1357-1422*	1204	*1175-1235*
Age-std	3066	*3015-3118*	2977	*2927-3027*	1462	*1428-1496*	1267	*1235-1299*

Table continued on next page

Table e7 continued

**Both**								
1-9	--	-	--	-	--	-	--	-
10-19	18	*12-28*	--	-	--	-	--	-
20-29	54	*42-71*	34	*25-48*	22	*15-33*	32	*23-45*
30-39	170	*149-195*	176	*154-201*	115	*98-136*	76	*62-93*
40-49	955	*909-1003*	786	*745-830*	498	*465-533*	330	*303-358*
50-54	2277	*2174-2385*	1665	*1578-1758*	1016	*948-1089*	714	*658-776*
55-59	3405	*3269-3547*	2528	*2412-2650*	1433	*1346-1525*	1124	*1048-1205*
60-64	4604	*4443-4771*	3209	*3077-3347*	1840	*1741-1945*	1646	*1553-1745*
65-69	5669	*5478-5867*	4006	*3850-4169*	2156	*2043-2275*	2167	*2054-2286*
70-74	7845	*7582-8117*	6035	*5812-6266*	3119	*2963-3285*	3153	*2997-3318*
75-79	9416	*9093-9750*	8361	*8070-8662*	4147	*3948-4357*	4167	*3970-4375*
80-84	9714	*9330-10,113*	11,092	*10,702-11,495*	5487	*5221-5766*	5456	*5194-5731*
85+	10,263	*9852-10,691*	17,065	*16,559-17,587*	7068	*6754-7396*	7113	*6801-7438*
No. cases	23,411		22,228		11,824		11,140	
Person yrs	9,913,623		10,089,058		10,175,528		10,225,569	
Crude	2361	*2331-2392*	2203	*2174-2232*	1162	*1141-1183*	1089	*1069-1110*
Age-std	2403	*2372-2434*	2173	*2144-2201*	1117	*1097-1137*	1042	*1023-1061*

Table continued on next page

Table e7 continued

Age (yrs)	Heart failure		Dilated cardiomyopathy		Hypertrophic cardiomyopathy		Cardiomyopathy, other	
**Females**	Rate*	*95% CI*	Rate*	*95% CI*	Rate*	*95% CI*	Rate*	*95% CI*
1-9	--	-	--	-	--	-	--	-
10-19	--	-	--	-	--	-	18	*10-33*
20-29	55	*38-81*	--	-	--	-	41	*26-64*
30-39	98	*76-127*	30	*19-47*	--	-	59	*43-82*
40-49	301	*266-341*	53	*39-71*	--	-	96	*77-119*
50-54	651	*576-735*	58	*39-87*	30	*17-53*	131	*100-172*
55-59	1122	*1016-1239*	103	*74-143*	43	*26-71*	215	*171-270*
60-64	1879	*1741-2029*	148	*113-195*	83	*57-119*	245	*199-303*
65-69	3088	*2901-3288*	183	*142-237*	87	*60-126*	352	*292-423*
70-74	6122	*5820-6440*	250	*195-320*	95	*64-142*	509	*428-605*
75-79	11,063	*10,619-11,525*	221	*166-293*	110	*74-164*	644	*546-760*
80-84	19,253	*18,585,19,944*	172	*120-246*	120	*78-184*	482	*389-597*
85+	37,791	*36,848-38,758*	125	*83-188*	146	*100-214*	597	*496-720*
No. cases	15,530		405		206		937	
Person yrs	5,105,261		5,173,311		5,174,524		5,170,448	
Crude	3042	*2994-3090*	78	*71-86*	40	*35-46*	181	*170-193*
Age-std	2641	*2599-2682*	72	*65-79*	36	*31-40*	164	*154-175*
**Males**								
1-9	--	-	--	-	--	-	--	-
10-19	23	*14-38*	--	-	--	-	32	*21-49*
20-29	68	*49-93*	20	*11-35*	--	-	48	*33-70*
30-39	163	*134-198*	47	*33-68*	29	*18-47*	68	*51-93*
40-49	633	*582-690*	153	*128-182*	61	*46-80*	185	*158-217*
50-54	1335	*1227-1453*	241	*197-294*	67	*46-98*	365	*311-429*
55-59	2377	*2221-2545*	315	*262-380*	79	*55-115*	460	*395-537*
60-64	3612	*3415-3821*	369	*310-440*	107	*78-148*	627	*548-716*
65-69	5779	*5513-6059*	492	*419-577*	81	*55-120*	836	*740-945*
70-74	9716	*9309-10,140*	558	*469-664*	114	*78-167*	938	*820-1073*
75-79	15,699	*15,110-16,310*	628	*522-754*	143	*97-210*	1314	*1157-1492*
80-84	26,582	*25,645-27,553*	585	*466-735*	158	*102-244*	1365	*1176-1585*
85+	47,233	*45,749,48,765*	526	*399-694*	--	-	1532	*1302-1803*
No. cases	16,484		1029		281		1797	
Person yrs	5,035,167		5,113,356		5,118,089		5,108,442	
Crude	3274	*3224-3324*	201	*189-214*	55	*49-62*	352	*336-368*
Age-std	3865	*3806-3924*	199	*187-212*	53	*47-60*	356	*340-373*

Table continued on next page

Table e7 continued

**Both**								
1-9	15	*8-26*	--	-	--	-	--	-
10-19	20	*13-30*	--	-	11	*7-19*	26	*18-36*
20-29	62	*49-79*	16	*10-26*	10	*6-19*	45	*34-60*
30-39	131	*112-153*	38	*29-51*	21	*14-31*	64	*51-80*
40-49	468	*436-501*	103	*89-119*	36	*28-47*	141	*124-160*
50-54	995	*928-1067*	150	*126-180*	49	*36-67*	249	*217-286*
55-59	1751	*1655-1852*	210	*178-246*	61	*45-83*	338	*298-384*
60-64	2733	*2612-2860*	258	*223-298*	95	*75-121*	434	*388-486*
65-69	4391	*4229-4560*	334	*292-382*	84	*64-110*	588	*531-651*
70-74	7808	*7557-8067*	396	*344-457*	104	*79-137*	713	*641-792*
75-79	13,137	*12,775-13,509*	406	*348-473*	125	*95-165*	949	*858-1049*
80-84	22,268	*21,715-22,836*	345	*285-419*	136	*100-185*	853	*754-964*
85+	40,944	*40,141-41,763*	261	*208-329*	118	*84-166*	915	*809-1034*
No. cases	32,014		1434		487		2734	
Person yrs	10,140,428		10,286,667		10,292,613		10,278,889	
Crude	3157	*3123-3192*	139	*132-147*	47	*43-52*	266	*256-276*
Age-std	3180	*3145-3214*	132	*125-139*	45	*41-49*	253	*243-262*

Table continued on next page

Table e7 continued

Age (yrs)	Multiple valve disorder		Non-rheumatic aortic valve disorder		Non-rheumatic mitral valve disorder		Rheumatic valve disorder	
Females	Rate	*95% CI*	Rate	*95% CI*	Rate	*95% CI*	Rate	*95% CI*
1-9	--	-	--	-	48	*31-76*	--	-
10-19	--	-	25	*15-41*	56	*40-79*	--	-
20-29	33	*20-54*	31	*19-51*	94	*71-126*	33	*20-54*
30-39	34	*22-53*	69	*51-93*	127	*101-158*	54	*38-76*
40-49	108	*88-133*	141	*118-169*	244	*213-280*	52	*38-69*
50-54	184	*147-232*	281	*233-338*	391	*334-457*	96	*70-132*
55-59	373	*314-443*	434	*370-509*	533	*461-615*	149	*114-196*
60-64	522	*452-604*	747	*662-844*	763	*677-861*	194	*153-246*
65-69	1006	*902-1122*	1458	*1332-1597*	1352	*1231-1486*	318	*262-386*
70-74	1920	*1756-2099*	2565	*2374-2772*	2084	*1912-2272*	657	*564-766*
75-79	3539	*3297-3799*	4240	*3972-4526*	3311	*3075-3564*	1076	*946-1223*
80-84	5354	*5019-5712*	6341	*5973-6733*	4000	*3710-4313*	1458	*1289-1649*
85+	8478	*8064-8913*	9583	*9136-10,051*	4997	*4680-5335*	1800	*1616-2005*
No. cases	4542		5487		4210		1348	
Person yrs	5,162,074		5,144,956		5,140,995		5,168,112	
Crude	880	*855-906*	1066	*1039-1095*	819	*795-844*	261	*247-275*
Age-std	733	*711-754*	907	*883-931*	718	*696-739*	225	*213-237*
**Males**								
1-9	--	-	53	*35-81*	29	*17-51*	--	-
10-19	21	*13-36*	46	*32-66*	34	*22-51*	--	-
20-29	43	*29-64*	43	*29-64*	37	*24-57*	--	-
30-39	33	*21-50*	67	*49-91*	98	*76-126*	37	*25-56*
40-49	138	*115-166*	140	*117-167*	202	*174-235*	52	*39-70*
50-54	261	*215-315*	390	*334-456*	395	*338-462*	97	*71-132*
55-59	474	*407-551*	646	*567-735*	677	*596-769*	170	*132-219*
60-64	788	*700-888*	1172	*1062-1292*	1084	*979-1200*	229	*184-286*
65-69	1369	*1244-1507*	2331	*2166-2509*	1713	*1572-1866*	397	*332-474*
70-74	2420	*2226-2632*	3696	*3453-3957*	2347	*2156-2556*	531	*444-634*
75-79	4056	*3772-4361*	5592	*5254-5953*	3711	*3439-4005*	968	*835-1122*
80-84	6435	*6004-6896*	8156	*7663-8681*	4802	*4430-5205*	1410	*1218-1634*
85+	9672	*9060-10,325*	12,300	*11,597-13,046*	5732	*5263-6242*	1999	*1734-2306*
No. cases	4121		5638		3880		1055	
Person yrs	5,107,705		5,086,375		5,093,399		5,115,694	
Crude	807	*783-832*	1108	*1080-1138*	762	*738-786*	206	*194-219*
Age-std	890	*863-917*	1232	*1200-1264*	813	*787-838*	221	*208-235*

Table continued on next page

Table e7 continued

**Both**								
1-9	--	-	40	*28-56*	39	*27-55*	17	*10-29*
10-19	15	*10-24*	36	*27-48*	45	*34-58*	12	*7-20*
20-29	38	*28-52*	37	*27-51*	64	*50-81*	25	*17-36*
30-39	33	*25-45*	68	*55-84*	112	*95-133*	46	*35-59*
40-49	123	*107-141*	141	*124-160*	223	*201-247*	52	*42-64*
50-54	223	*192-258*	336	*298-379*	393	*352-439*	96	*77-120*
55-59	423	*378-475*	540	*488-597*	605	*550-666*	160	*133-192*
60-64	654	*597-717*	957	*887-1033*	922	*853-997*	212	*180-249*
65-69	1184	*1102-1272*	1884	*1779-1995*	1529	*1435-1629*	356	*312-406*
70-74	2158	*2030-2293*	3101	*2946-3264*	2209	*2079-2347*	597	*532-671*
75-79	3774	*3587-3971*	4853	*4639-5077*	3493	*3313-3683*	1027	*932-1131*
80-84	5808	*5540-6089*	7100	*6800-7413*	4337	*4105-4582*	1438	*1308-1580*
85+	8883	*8537-9243*	10,502	*10,120-10,899*	5246	*4980-5526*	1868	*1714-2036*
No. cases	8663		11,125		8090		2403	
Person yrs	10,269,779		10,231,331		10,234,394		10,283,807	
Crude	844	*826-861*	1087	*1067-1108*	790	*773-808*	234	*225-243*
Age-std	803	*786-819*	1050	*1031-1070*	759	*742-776*	223	*214-231*

Table continued on next page

Table e7 continued

Age (yrs)	Atrioventricular block, 2nd degree		Atrioventricular block, 3rd degree		Bifascicular block		Pericardial effusion		Supraventricular tachycardia		Trifascicular block		Ventricular tachycardia	
**Females**	Rate	*95% CI*	Rate	*95% CI*	Rate	*95% CI*	Rate	*95% CI*	Rate	*95% CI*	Rate	*95% CI*	Rate	*95% CI*
1-9	--	-	--	-	--	-	--	-	36	*21-60*	--	-	--	-
10-19	--	-	--	-	--	-	23	*14-39*	140	*113-173*	--	-	20	*11-35*
20-29	--	-	--	-	--	-	25	*14-43*	282	*238-333*	--	-	27	*15-46*
30-39	20	*11-35*	--	-	--	-	54	*38-76*	286	*247-332*	--	-	44	*30-65*
40-49	26	*17-40*	23	*15-36*	--	-	93	*75-117*	396	*356-441*	--	-	52	*38-69*
50-54	35	*21-60*	35	*21-60*	--	-	172	*135-218*	427	*367-497*	--	-	91	*66-126*
55-59	54	*35-85*	57	*37-89*	--	-	241	*194-298*	609	*532-697*	--	-	120	*89-163*
60-64	88	*62-126*	103	*74-142*	--	-	265	*216-325*	642	*563-732*	--	-	145	*111-191*
65-69	124	*91-169*	196	*153-251*	--	-	463	*395-544*	789	*697-892*	--	-	171	*131-223*
70-74	246	*192-316*	409	*337-496*	--	-	600	*511-704*	1095	*972-1233*	--	-	302	*241-378*
75-79	400	*324-494*	575	*483-686*	78	*48-125*	800	*690-928*	1430	*1278-1600*	64	*38-108*	446	*366-545*
80-84	643	*534-773*	885	*756-1036*	132	*87-198*	843	*717-991*	1595	*1417-1796*	109	*69-170*	482	*389-597*
85+	969	*836-1122*	1743	*1562-1946*	380	*300-480*	886	*760-1032*	1600	*1426-1796*	396	*315-498*	571	*471-691*
No. cases	589		872		135		1175		2731		129		641	
Person yrs	5,173,166		5,171,581		5,175,911		5,171,892		5,138,482		5,175,832		5,171,827	
Crude	114	*105-123*	169	*158-180*	26	*22-31*	227	*215-241*	531	*512-552*	25	*21-30*	124	*115-134*
Age-std	96	*88-104*	140	*130-149*	21	*17-24*	203	*191-214*	490	*472-509*	19	*16-23*	110	*102-119*
**Males**														
1-9	--	-	--	-	--	-	--	-	70	*49-101*	--	-	--	-
10-19	--	-	--	-	--	-	--	-	126	*101-156*	--	-	17	*9-30*
20-29	--	-	--	-	--	-	32	*20-51*	157	*128-194*	--	-	--	-
30-39	--	-	21	*21-36*	--	-	54	*38-76*	153	*125-188*	--	-	46	*31-66*
40-49	31	*21-46*	26	*17-40*	--	-	105	*85-129*	234	*204-270*	--	-	104	*84-128*
50-54	92	*66-127*	79	*56-112*	--	-	169	*133-214*	378	*323-444*	--	-	154	*120-197*
55-59	105	*76-145*	108	*78-148*	--	-	227	*182-282*	561	*488-645*	--	-	284	*233-345*
60-64	183	*143-234*	250	*202-308*	44	*26-72*	314	*260-379*	710	*626-806*	46	*28-76*	503	*433-584*
65-69	325	*267-395*	371	*308-445*	97	*68-139*	475	*404-558*	905	*804-1018*	81	*55-120*	541	*464-630*
70-74	583	*492-691*	685	*585-801*	210	*158-279*	592	*500-701*	1178	*1045-1328*	210	*158-279*	844	*733-973*
75-79	1068	*927-1229*	1474	*1307-1661*	329	*256-424*	841	*718-985*	1533	*1362-1725*	472	*382-583*	1120	*976-1286*
80-84	1341	*1153-1559*	2008	*1774-2271*	560	*444-707*	964	*807-1151*	1557	*1353-1793*	790	*649-961*	1430	*1236-1655*
85+	2231	*1948-2554*	3768	*3394-4183*	1265	*1058-1513*	1107	*914-1340*	2028	*1758-2338*	2052	*1783-2362*	1526	*1296-1797*
No. cases	988		1344		359		1075		2281		479		1357	
Person yrs	5,116,015		5,113,725		5,119,770		5,116,182		5,094,729		5,119,186		5,112,235	
Crude	193	*181-206*	263	*249-277*	70	*63-78*	210	*198-223*	448	*430-466*	94	*86-102*	265	*252-280*
Age-std	212	*198-225*	295	*279-310*	80	*72-88*	216	*203-229*	459	*440-478*	109	*100-119*	275	*260-290*

Table continued on next page

Table e7 continued

**Both**														
1-9	--	-	--	-	--	-	22	*14-36*	53	*40-72*	--	-	--	-
10-19	--	-	--	-	--	-	19	*13-29*	132	*114-154*	--	-	18	*12-28*
20-29	13	*8-23*	--	-	--	-	29	*20-41*	215	*189-245*	--	-	22	*15-33*
30-39	17	*11-26*	16	*11-25*	--	-	54	*42-69*	220	*195-248*	--	-	45	*34-59*
40-49	29	*22-38*	24	*18-33*	--	-	99	*85-116*	315	*289-343*	--	-	78	*65-92*
50-54	64	*48-84*	58	*43-77*	--	-	170	*144-201*	402	*361-449*	--	-	123	*101-150*
55-59	80	*61-104*	83	*64-107*	16	*9-28*	234	*201-272*	585	*531-644*	--	-	202	*172-239*
60-64	135	*110-165*	176	*147-210*	26	*16-41*	289	*252-332*	676	*617-740*	29	*19-45*	323	*283-368*
65-69	222	*188-262*	281	*243-326*	57	*41-79*	469	*418-526*	845	*776-921*	51	*36-72*	352	*308-401*
70-74	406	*353-468*	540	*478-610*	116	*90-151*	596	*531-669*	1134	*1042-1234*	121	*93-156*	559	*496-630*
75-79	704	*626-791*	984	*891-1086*	192	*154-241*	819	*735-913*	1477	*1361-1602*	250	*205-304*	753	*672-843*
80-84	936	*832-1052*	1355	*1230-1494*	312	*255-382*	894	*793-1008*	1579	*1442-1729*	395	*330-473*	879	*779-992*
85+	1396	*1264-1542*	2428	*2251-2619*	680	*590-784*	961	*852-1083*	1746	*1596-1910*	957	*849-1079*	895	*790-1013*
No. cases	1577		2216		494		2250		5012		608		1998	
Person yrs	10,289,181		10,285,306		10,295,681		10,288,074		10,233,210		10,295,018		10,284,062	
Crude	153	*146-161*	215	*207-225*	48	*44-52*	219	*210-228*	490	*476-504*	59	*55-64*	194	*186-203*
Age-std	145	*138-153*	205	*196-213*	45	*41-49*	208	*200-217*	472	*459-485*	55	*51-60*	185	*176-193*

Table continued on next page

Table e7 continued

Age (years)	Ischemic stroke		Stroke, not otherwise specified	
**Females**	Rate*	*95% CI*	Rate*	*95% CI*
1-9	--	-	--	-
10-19	--	-	--	-
20-29	43	*28-66*	29	*17-48*
30-39	100	*78-129*	89	*68-116*
40-49	252	*220-288*	217	*188-251*
50-54	468	*405-541*	451	*389-522*
55-59	687	*605-780*	599	*522-686*
60-64	955	*858-1063*	934	*838-1041*
65-69	1605	*1472-1750*	1311	*1191-1443*
70-74	3029	*2820-3253*	2138	*1963-2328*
75-79	5114	*4819-5427*	3577	*3331-3841*
80-84	8074	*7656-8515*	5206	*4871-5563*
85+	14,835	*14,276-15,417*	8634	*8206-9085*
No. cases	7370		5031	
Person yrs	5,140,505		5,133,139	
Crude	1434	*1401-1467*	980	*953-1008*
Age-std	1209	*1181-1236*	843	*819-866*
**Males**				
1-9	--	-	--	-
10-19	18	*10-32*	--	-
20-29	21	*12-38*	--	-
30-39	109	*86-139*	46	*31-66*
40-49	344	*306-386*	227	*197-261*
50-54	694	*617-781*	487	*424-561*
55-59	1361	*1244-1488*	869	*777-972*
60-64	1945	*1802-2098*	1316	*1200-1444*
65-69	2917	*2731-3116*	1832	*1685-1991*
70-74	4545	*4273-4834*	2937	*2720-3171*
75-79	6780	*6405-7176*	4038	*3751-4347*
80-84	9521	*8986-10,088*	5456	*5054-5890*
85+	14,164	*13,405-14,966*	8216	*7640-8836*
No. cases	7299		4487	
Person yrs	5,079,405		5,079,287	
Crude	1437	*1404-1470*	883	*858-910*
Age-std	1573	*1537-1610*	963	*935-991*

Table continued on next page

Table e7 continued

**Both**				
1-9	--	-	17	*10-29*
10-19	18	*12-27*	--	-
20-29	31	*22-44*	21	*14-32*
30-39	105	*88-124*	67	*54-83*
40-49	298	*273-325*	222	*200-246*
50-54	582	*532-638*	469	*424-519*
55-59	1025	*953-1103*	735	*674-801*
60-64	1444	*1357-1537*	1123	*1047-1205*
65-69	2244	*2129-2365*	1565	*1469-1666*
70-74	3745	*3575-3925*	2516	*2376-2663*
75-79	5867	*5631-6113*	3786	*3597-3985*
80-84	8678	*8344-9024*	5310	*5051-5584*
85+	14,608	*14,155-15,076*	8492	*8146-8854*
No. cases	14,669		9518	
Person yrs	10,219,909		10,212,426	
Crude	1435	*1412-1459*	932	*913-951*
Age-std	1384	*1362-1407*	902	*883-920*

Table continued on next page

Table e7 continued

Age (yrs)	Atrial fibrillation		Atrioventricular block, 1st degree		Left bundle branch block		Right bundle branch block		Sick sinus syndrome	
**Females**	Rate*	*95% CI*	Rate*	*95% CI*	Rate*	*95% CI*	Rate*	*95% CI*	Rate*	*95% CI*
1-9	--	-	--	-	--	-	--	-	--	-
10-19	22	*13-37*	--	-	--	-	50	*35-71*	--	-
20-29	66	*46-93*	--	-	--	-	86	*64-117*	--	-
30-39	138	*111-171*	25	*15-41*	36	*24-55*	77	*58-103*	--	-
40-49	409	*368-455*	38	*27-54*	96	*77-119*	122	*101-148*	13	*7-24*
50-54	842	*756-938*	61	*41-90*	192	*153-240*	200	*160-249*	33	*19-57*
55-59	1751	*1617-1896*	86	*60-123*	347	*290-415*	264	*215-342*	40	*24-68*
60-64	3066	*2887-3256*	168	*130-217*	534	*463-617*	340	*284-407*	68	*46-102*
65-69	5472	*5218-5738*	311	*255-378*	748	*659-849*	560	*484-648*	140	*104-187*
70-74	10,402	*10,000-10,820*	632	*541-738*	1477	*1334-1635*	855	*748-978*	322	*259-400*
75-79	18,384	*17,794-18,993*	1068	*939-1215*	2331	*2136-2544*	1573	*1415-1749*	594	*500-706*
80-84	28,473	*27,629-29,342*	2073	*1869-2298*	3866	*3583-4171*	2596	*2367-2847*	817	*693-963*
85+	47,991	*46,880-49-128*	4308	*4017-4620*	7054	*6677-7452*	5248	*4926-5591*	1063	*924-1223*
No. cases	21,509		1809		3548		2658		675	
Person yrs	5,051,596		5,170,099		5,162,356		5,166,741		5,171,954	
Crude	4258	*4201-4315*	350	*334-366*	687	*665-710*	514	*495-534*	131	*121-141*
Age-std	3911	*3859-3963*	283	*270-296*	569	*550-588*	429	*412-445*	110	*102-118*
**Males**										
1-9	--	-	--	-	--	-	--	-	--	-
10-19	37	*25-55*	--	-	--	-	72	*54-96*	--	-
20-29	168	*137-205*	--	-	20	*11-35*	100	*77-130*	--	-
30-39	331	*289-380*	24	*15-41*	36	*24-54*	127	*102-159*	--	-
40-49	816	*757-879*	67	*51-87*	153	*128-182*	211	*182-245*	15	*9-27*
50-54	1990	*1856-2133*	117	*88-155*	325	*274-386*	392	*336-459*	42	*26-68*
55-59	3409	*3220-3610*	249	*202-307*	443	*378-518*	577	*502-662*	59	*39-91*
60-64	5935	*5679-6203*	474	*406-552*	722	*637-817*	891	*796-997*	122	*90-165*
65-69	10,511	*10,144-10,892*	817	*722-924*	1246	*1127-1378*	1466	*1337-1608*	211	*166-269*
70-74	17,111	*16,555-17,686*	1606	*1449-1779*	2340	*2149-2547*	2455	*2259-2667*	351	*282-437*
75-79	25,627	*24,841-26,438*	2974	*2733-3236*	3894	*3616-4194*	4158	*3870-4467*	765	*648-903*
80-84	39,335	*38,129-40,580*	5082	*4701-5493*	5793	*5385-6232*	6217	*5794-6672*	1022	*860-1215*
85+	59,245	*57-472-61,073*	8788	*8204-9413*	10,355	*9719-11,032*	11,182	*10,520-11,886*	1241	*1035-1487*
No. cases	23,566		2984		3991		4589		636	
Person yrs	4,965,632		5,110,026		5,105,869		5,102,613		5,116,543	
Crude	4746	*4686-4807*	584	*563-605*	782	*758-806*	899	*874-926*	124	*115-134*
Age-std	5824	*5749-5898*	660	*636-683*	869	*842-896*	992	*963-1020*	135	*125-146*

Table continued on next page

Table e7 continued

**Both**					
1-9	-- -	-- -	-- -	16 *9-28*	-- -
10-19	29 *21-41*	-- -	-- -	61 *49-77*	-- -
20-29	120 *101-143*	18 *12-28*	12 *7-21*	94 *77-114*	13 *8-23*
30-39	235 *209-264*	25 *17-35*	36 *27-48*	102 *86-122*	11 *6-18*
40-49	613 *576-651*	53 *43-65*	124 *109-142*	167 *148-188*	14 *10-21*
50-54	1419 *1339-1505*	89 *70-112*	259 *226-297*	297 *261-337*	38 *26-54*
55-59	2579 *2462-2702*	168 *140-201*	395 *351-444*	421 *375-472*	50 *36-69*
60-64	4472 *4316-4634*	319 *280-364*	627 *571-689*	613 *557-674*	95 *75-121*
65-69	7893 *7672-8121*	558 *503-620*	991 *916-1072*	1002 *927-1084*	175 *145-511*
70-74	13,514 *13,176-13,861*	1094 *1004-1192*	1886 *1767-2014*	1613 *1504-1732*	335 *287-392*
75-79	21-572 *21,091-22,063*	1934 *1802-2075*	3041 *2874-3218*	2745 *2586-2913*	672 *595-757*
80-84	32,847 *32-144-33,566*	3331 *3130-3544*	4673 *4434-4926*	4108 *3885-4345*	903 *802-1017*
85+	51,646 *50,697-52,613*	5815 *5537-6107*	8171 *7839-8518*	7242 *6931-7568*	1123 *1005-1255*
No. cases	45,075	4793	7539	7247	1311
Person yrs	10,017,228	10,280,126	10,268,225	10,269,354	10,288,497
Crude	4500 *4458-4541*	466 *453-480*	734 *718-751*	706 *690-722*	127 *121-135*
Age-std	4756 *4712-4800*	442 *429-454*	698 *682-714*	671 *656-687*	121 *115-128*

Table continued on next page

Table e7 continued

Age (yrs)	TIA		Intracerebral hemorrhage		Subarachnoid hemorrhage		Subdural hematoma	
**Females**	Rate*	*95% CI*	Rate*	*95% CI*	Rate*	*95% CI*	Rate*	*95% CI*
1-9	--	-	--	-	--	-	--	-
10-19	--	-	--	-	--	-	--	-
20-29	41	*26-64*	--	-	33	*20-54*	--	-
30-39	135	*108-167*	33	*21-51*	62	*45-86*	--	-
40-49	354	*316-397*	69	*54-90*	119	*97-145*	22	*14-34*
50-54	748	*668-839*	144	*111-187*	215	*174-266*	28	*15-50*
55-59	1088	*984-1204*	204	*161-257*	187	*146-238*	52	*32-82*
60-64	1648	*1519-1789*	237	*191-294*	226	*181-282*	88	*62-126*
65-69	2087	*1933-2252*	401	*338-477*	246	*197-307*	93	*65-133*
70-74	3773	*3537-4025*	740	*641-855*	343	*277-423*	206	*157-271*
75-79	5751	*5433-6088*	1046	*919-1192*	401	*325-495*	349	*279-437*
80-84	7642	*7227-8080*	1686	*1504-1891*	580	*477-705*	619	*512-747*
85+	9972	*9502-10,466*	2677	*2450-2925*	619	*516-744*	951	*820-1102*
No. cases	7293		1634		858		536	
Person yrs	5,104,151		5,168,503		5,167,153		5,174,219	
Crude	1429	*1396-1462*	316	*301-332*	166	*155-178*	104	*95-113*
Age-std	1270	*1241-1300*	266	*253-279*	148	*138-158*	86	*79-93*
**Males**								
1-9	--	-	--	-	--	-	--	-
10-19	--	-	--	-	20	*12-34*	--	-
20-29	25	*15-42*	21	*12-38*	34	*22-53*	--	-
30-39	99	*77-128*	37	*25-56*	67	*49-91*	--	-
40-49	395	*355-440*	110	*89-135*	118	*97-144*	29	*19-43*
50-54	882	*795-979*	204	*164-253*	139	*107-181*	45	*28-71*
55-59	1601	*1473-1739*	287	*236-348*	176	*137-226*	74	*50-108*
60-64	2343	*2186-2512*	410	*348-484*	175	*136-225*	110	*80-152*
65-69	3366	*3164-3580*	694	*607-793*	273	*221-339*	237	*188-298*
70-74	5039	*4749-5347*	840	*729-968*	233	*178-304*	394	*321-485*
75-79	7000	*6612-7410*	1474	*1307-1662*	181	*129-255*	659	*551-789*
80-84	8989	*8456-9555*	2155	*1913-2427*	387	*293-512*	1417	*1224-1640*
85+	10,588	*9911-11,310*	2886	*2562-3250*	516	*390-683*	2317	*2030-2646*
No. cases	7319		1679		621		805	
Person yrs	5,046,705		5,111,299		5,113,502		5,117,208	
Crude	1450	*1417-1484*	328	*313-345*	121	*112-131*	157	*147-169*
Age-std	1588	*1552-1624*	348	*332-365*	121	*111-130*	175	*163-187*

Table continued on next page

Table e7 continued

**Both**								
1-9	--	-	--	-	--	-	--	-
10-19	14	*9-23*	14	*9-23*	16	*10-25*	--	-
20-29	32	*23-45*	18	*12-28*	33	*24-47*	12	*7-21*
30-39	117	*99-138*	35	*26-47*	65	*52-80*	13	*8-21*
40-49	375	*347-405*	90	*76-105*	118	*103-136*	25	*19-34*
50-54	816	*755-881*	174	*147-206*	177	*150-208*	36	*25-52*
55-59	1345	*1262-1434*	245	*211-285*	181	*152-216*	63	*47-84*
60-64	1992	*1889-2100*	323	*283-368*	200	*170-237*	99	*78-126*
65-69	2709	*2582-2842*	544	*489-605*	259	*223-303*	164	*135-198*
70-74	4370	*4183-4565*	788	*712-871*	290	*246-343*	296	*251-349*
75-79	6313	*6064-6573*	1241	*1136-1355*	301	*251-360*	490	*426-564*
80-84	8200	*7869-8545*	1883	*1734-2045*	499	*425-585*	954	*850-1071*
85+	10,179	*9790-10,583*	2748	*2560-2950*	584	*501-681*	1414	*1281-1561*
No. cases	14,612		3313		1479		1341	
Person yrs	10,150,856		10,279,802		10,280,655		10,291,428	
Crude	1439	*1416-1463*	322	*311-333*	144	*137-151*	130	*124-137*
Age-std	1418	*1395-1441*	304	*294-315*	136	*129-143*	123	*116-129*

Table continued on next page

Table e7 continued

Age (yrs)	Hypertension		Primary pulmonary hypertension		Secondary pulmonary hypertension		Raynauds	
**Females**	Rate*	*95% CI*	Rate*	*95% CI*	Rate*	*95% CI*	Rate*	*95% CI*
1-9	104	*77-142*	--	-	--	-	66	*45-97*
10-19	161	*132-197*	--	-	--	-	1112	*1030-1199*
20-29	951	*868-1042*	--	-	25	*14-43*	1045	*957-1140*
30-39	2927	*2793-3068*	20	*11-35*	21	*12-37*	1279	*1191-1372*
40-49	8543	*8339-8752*	59	*44-78*	53	*39-71*	1145	*1074-1221*
50-54	14,299	*13,901-14,709*	93	*68-129*	104	*76-141*	921	*830-1021*
55-59	18,074	*17,574-18,589*	126	*94-169*	135	*101-179*	941	*844-1049*
60-64	23,227	*22,626-23,845*	200	*158-252*	197	*155-249*	1053	*950-1167*
65-69	31,497	*30,710-32,304*	398	*335-473*	236	*188-295*	1142	*1030-1266*
70-74	43,947	*42,778-45,147*	704	*608-816*	575	*489-677*	1212	*1083-1357*
75-79	55,416	*53,826-57,053*	1064	*936-1211*	877	*761-1011*	1278	*1135-1439*
80-84	65,352	*63,211-67,566*	1680	*1498-1884*	1294	*1136-1474*	1219	*1065-1396*
85+	70,919	*68,606-73,310*	2237	*2030-2464*	1600	*1428-1794*	755	*639-893*
No. cases	46,949		1464		1163		5234	
Person yrs	4,185,552		5,169,913		5,174,950		5,107,807	
Crude	11,217	*11,116-11,319*	283	*269-298*	225	*212-238*	1025	*997-1053*
Age-std	14,856	*14,722-14,991*	239	*226-251*	191	*180-202*	1000	*973-1027*
**Males**								
1-9	124	*94-163*	--	-	--	-	39	*24-63*
10-19	171	*142-205*	--	-	--	-	377	*332-427*
20-29	739	*671-813*	--	-	--	-	285	*244-332*
30-39	3160	*3021-3305*	21	*12-36*	18	*10-32*	345	*301-395*
40-49	10,693	*10,464-10,927*	48	*35-65*	41	*29-57*	462	*418-511*
50-54	19,228	*18,761-19,706*	60	*40-89*	57	*38-86*	569	*499-647*
55-59	25,988	*25,347-26,617*	179	*139-229*	91	*64-128*	699	*617-793*
60-64	34,177	*33,412-34,961*	209	*166-263*	165	*128-214*	893	*799-1000*
65-69	42,502	*41,524-43,502*	422	*356-502*	269	*217-334*	791	*697-898*
70-74	49,796	*48,463-51,166*	763	*658-885*	521	*435-623*	939	*821-1074*
75-79	58,435	*56,670-60,256*	1073	*932-1234*	845	*721-989*	939	*808-1092*
80-84	65,991	*63,610-68,461*	1639	*1431-1879*	1515	*1315-1745*	1079	*912-1278*
85+	75,028	*72,043-78,135*	2105	*1832-2419*	2021	*1754-2328*	852	*685-1061*
No. cases	52,790		1132		911		2632	
Person yrs	4,210,136		5,115,818		5,119,777		5,090,552	
Crude	12,539	*12,432-12-646*	221	*209-235*	178	*167-190*	517	*498-537*
Age-std	17,820	*17,668-17,972*	239	*225-253*	194	*182-207*	506	*487-525*

Table continued on next page

Table e7 continued

**Both**								
1-9	114	*93-140*	--	-	--	-	52	*39-71*
10-19	166	*145-190*	--	-	--	-	729	*683-778*
20-29	837	*783-895*	14	*9-24*	16	*10-26*	637	*590-687*
30-39	3044	*2947-3145*	20	*14-30*	20	*13-29*	808	*759-860*
40-49	9614	*9460-9770*	53	*43-65*	47	*37-58*	801	*759-845*
50-54	16,745	*16,438-17,059*	76	*59-98*	80	*63-102*	742	*685-805*
55-59	21,949	*21,553-22,352*	152	*126-184*	112	*90-140*	819	*754-889*
60-64	28,451	*27,967-28,943*	204	*173-241*	181	*152-216*	974	*902-1050*
65-69	36,637	*36,015-37,270*	410	*363-463*	252	*216-295*	970	*895-1050*
70-74	46,670	*45,786-47,571*	732	*659-813*	549	*487-620*	1082	*992-1180*
75-79	56,807	*55,620-58,019*	1068	*971-1175*	862	*776-958*	1123	*1023-1233*
80-84	65,639	*64,039-67,279*	1663	*1523-1815*	1387	*1260-1526*	1160	*1044-1290*
85+	72,509	*70,670-74,395*	2192	*2025-2373*	1743	*1595-1905*	788	*690-900*
No. cases	99,739		2596		2074		7866	
Person yrs	8,395,688		10,285,731		10,294,728		10,198,358	
Crude	11,880	*11,806-11,954*	252	*243-262*	201	*193-210*	771	*754-789*
Age-std	16,268	*16,167-16,369*	239	*230-249*	190	*182-199*	748	*731-765*

Table continued on next page

Table e7 continued

Age (yrs)	Pulmonary embolism		VTE (excluding PE)		Abdominal aortic aneurysm		Peripheral arterial disease	
**Females**	Rate*	*95% CI*	Rate*	*95% CI*	Rate*	*95% CI*	Rate*	*95% CI*
1-9	--	-	--	-	--	-	--	-
10-19	46	*32-67*	81	*61-108*	--	-	37	*24-55*
20-29	273	*230-324*	366	*316-424*	--	-	45	*30-69*
30-39	294	*254-341*	449	*399-506*	--	-	82	*62-108*
40-49	437	*394-484*	637	*585-694*	25	*16-39*	223	*193-258*
50-54	587	*516-668*	813	*728-907*	53	*35-81*	517	*451-593*
55-59	730	*646-826*	901	*806-1007*	72	*48-106*	657	*577-748*
60-64	1079	*975-1194*	1281	*1167-1407*	145	*111-191*	1140	*1033-1257*
65-69	1818	*1676-1973*	1946	*1798-2107*	298	*244-365*	1655	*1519-1803*
70-74	2298	*2116-2494*	2488	*2298-2694*	792	*689-910*	2356	*2171-2555*
75-79	3305	*3069-3558*	3371	*3132-3629*	1288	*1146-1449*	3824	*3569-4098*
80-84	3853	*3569-4159*	4086	*3791-4404*	1893	*1699-2109*	4868	*4544-5215*
85+	4361	*4066-4677*	5242	*4914-5592*	2270	*2061-2499*	6503	*6134-6893*
No. cases	4853		5629		1447		4964	
Person yrs	5,136,702		5,103,956		5,169,422		5,129,243	
Crude	945	*919-972*	1103	*1074-1132*	280	*266-295*	968	*941-995*
Age-std	845	*821-869*	996	*970-1022*	235	*223-247*	846	*823-870*
**Males**								
1-9	--	-	--	-	--	-	--	-
10-19	--	-	35	*23-53*	--	-	20	*12-34*
20-29	116	*91-148*	250	*212-295*	21	*12-38*	39	*26-60*
30-39	223	*189-264*	451	*401-507*	--	-	98	*76-126*
40-49	408	*367-453*	696	*642-755*	46	*34-64*	337	*300-379*
50-54	742	*662-831*	999	*905-1102*	107	*79-144*	1065	*969-1171*
55-59	929	*834-1036*	1360	*1243-1488*	272	*223-332*	1838	*1701-1986*
60-64	1458	*1335-1592*	1794	*1657-1942*	1371	*1252-1500*	2697	*2527-2878*
65-69	1913	*1764-2075*	2307	*2141-2485*	3001	*2813-3202*	3789	*3574-4017*
70-74	2606	*2403-2826*	2705	*2497-2930*	3052	*2832-3289*	5280	*4983-5595*
75-79	3385	*3126-3666*	3352	*3093-3634*	4485	*4184-4808*	6557	*6183-6953*
80-84	3900	*3567-4264*	4085	*3741-4460*	6164	*5738-6621*	8242	*7737-8779*
85+	4401	*3993-4850*	4467	*4053-4924*	7695	*7146-8287*	9335	*8714-10,000*
No. cases	4334		5293		4524		7518	
Person yrs	5,091,009		5,068,245		5,095,970		5,049,641	
Crude	851	*826-877*	1044	*1017-1073*	888	*862-914*	1489	*1456-1523*
Age-std	881	*855-907*	1069	*1040-1098*	964	*936-992*	1598	*1562-1635*

Table continued on next page

Table e7 continued

**Both**								
1-9	--	-	--	-	--	-	--	-
10-19	29	*21-41*	57	*46-72*	--	-	28	*20-39*
20-29	189	*164-217*	304	*272-339*	13	*8-23*	42	*31-56*
30-39	259	*232-289*	450	*414-489*	15	*9-23*	90	*75-108*
40-49	422	*392-455*	667	*629-707*	36	*28-46*	280	*256-307*
50-54	665	*611-725*	907	*843-976*	80	*63-102*	793	*734-857*
55-59	830	*765-901*	1132	*1056-1214*	172	*144-206*	1249	*1168-1334*
60-64	1267	*1186-1354*	1536	*1445-1631*	752	*690-819*	1907	*1806-2013*
65-69	1865	*1760-1975*	2123	*2011-2241*	1613	*1516-1715*	2687	*2561-2820*
70-74	2444	*2307-2589*	2591	*2449-2742*	1858	*1739-1985*	3724	*3552-3904*
75-79	3342	*3165-3528*	3363	*3185-3551*	2727	*2569-2895*	5047	*4826-5277*
80-84	3873	*3654-4104*	4086	*3859-4325*	3656	*3444-3881*	6257	*5973-6555*
85+	4375	*4133-4630*	4978	*4717-5254*	4072	*3840-4318*	7446	*7122-7785*
No. cases	9187		10,922		5971		12,482	
Person yrs	10,227,711		10,172,201		10,265,391		10,178,884	
Crude	898	*880-917*	1074	*1054-1094*	582	*567-597*	1226	*1205-1248*
Age-std	861	*844-879*	1035	*1015-1054*	555	*541-570*	1190	*1169-1211*

*Rates are given per 1,000,000 person years. Cases and person years are numbers; all other figures are rates.
Abbreviations – yrs: years; CI: confidence interval; No.cases: total number of cases; Person yrs: Total person-years; Age-std: age-standardized

References

[1] Jones DA, Candio P, Shakir R, Ntentas G, Ramroth J, Gray AM, et al. Informing radiotherapy decisions in stage I/IIa Hodgkin lymphoma: modeling life expectancy using radiation dosimetry. Blood Adv 2022;6:909–19. doi:10.1182/bloodadvances.2021006254.

[2] Cutter DJ, Ramroth J, Diez P, Buckle A, Ntentas G, Popova B, et al. Predicted risks of cardiovascular disease following chemotherapy and radiotherapy in the UK NCRI RAPID trial of positron emission tomography-directed therapy for early-stage Hodgkin lymphoma. J Clin Oncol 2021;39:3591–601. doi:10.1200/JCO.21.00408.

[3] WHO mortality statistics n.d. http://apps.who.int/healthinfo/statistics/mortality/causeofdeath_query/ (accessed November 15, 2017).
